# Abstracts of papers presented at the 1999 Pittsburgh Conference

**DOI:** 10.1155/S1463924600000018

**Published:** 2000


Journal of Automated Methods & Management in Chemistry, Vol. 22, No. 1 (January–February 2000) pp. 1–28

### Abstracts of papers presented at the 1999 Pittsburgh Conference

The following 79 abstracts form Part C of three issues of *Journal of Automated Methods & Management in Chemistry* devoted to abstracts of papers presented at the 50th Anniversary Pittcon held in March 1999 in New Orleans, LA. The editors have selected, from over 1000 presentations, those of particular interest to the *Journal of Automatic Chemistry's* readers. For information on this year's Pittcon, contact The Pittsburgh Conference, 300 Penn Center Boulevard, Suite 332, Pittsburgh, PA 15235–5503, USA. Tel.: 412 825 3220; fax.: 412 825 3224; World Wide Web: http://www.pittcon.org.

### The hand-held electronic nose: providing answers where they are needed

*Beth Munoz, Greg Steinthal and Steven Sunshine, Cyrano Sciences, Pasadena, CA 91107, USA*

Array-based sensing technology is emerging as a powerful chemical analysis tool. The use of conducting polymer composites as sensing elements has resulted in a simple device that can easily identify chemicals and/or provide information about batch to batch chemical consistency. This paper will focus on the technology, performance and use of the electronic nose to provide solutions to industrial problems.

In the laboratory, we have demonstrated that the electronic nose can solve a variety of industrial problems. One problem involves the verification of incoming raw materials in a chemical plant. The second problem involves the use of the electronic nose to monitor on-line the addition of an active component to a formulation. Both problems are of considerable importance in a variety of industries. Currently, the technologies used to address these problems are cumbersome and expensive. A hand-held electronic nose provides ease of use at a lower cost.

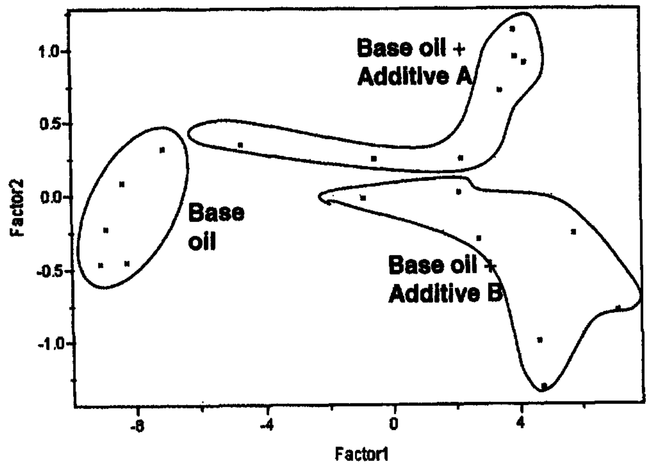

*Results from principal component analysis of a 32-sensor array to a base oil and the base oil plus additives.*

### Considerations for automatic, fast in-line dilution in atomic spectrometry

*Stuart Baker, Steve Morton and Mike Wassall, Unicam Atomic Absorption, PO Box 207, York Street, Cambridge CB1 2SU, UK*

Because of the inherently small dynamic working range in atomic absorption spectrometry, many varied steps have been taken to improve this limitation. Limited by the inherent noise of the technique at low levels and calibration non-linearity at high levels, the approaches have concentrated primarily on improving one, or both, of these factors. Such improvements have now been largely maximized and hence there is a growing interest in returning to attempts to automate what an analyst must do in the face of such limitations, i.e. dilution.

This paper will review alternatives available in providing such automatic in-line dilution and in the process assess each against the necessary demanding specification requirements. The requirements are derived primarily from the perspective of non-degradation of the fundamental performance characteristics of the AAS technique. From this treatment, a new approach to dilution is derived and comprehensively evaluated against the initial requirement in a typical laboratory situation.

Fundamental performance characteristics are presented, including carry-over, precision and absolute accuracy in varying situations. In addition, a figure of merit is derived for the analytical speed of the device against alternative methods.

In order to assess the performance with varying sample and matrix type, results are presented for the dilution performance with a wide range of typical analytical solutions, including organic solvents. The results indicate that the approach is successful in automating a skilled and time-consuming problem, and greatly enhances both the ease and application of the AAS technique.

### Determination of lead by flow injection hydride generation atomic absorption spectrometry

*Julian F. Tyson, Robert I. Ellis, Cesar Vargas, Nusret Ertas, Zikri Arslan, Latif Elci, Susan McIntosh[1] and Frank Fernandez[1], Department of Chemistry, Box 34510, University of Massachusetts, Amherst, MA 01001-4510, USA; [1]Perkin-Elmer, 761 Main Avenue, Norwalk, CT 06859, USA*

Flow injection, hydride generation atomic absorption spectrometry procedures for the determination of total lead in a variety of matrices have been developed. Ferricyanide is used as an oxidant (to produce lead in the +4 oxidation state) but no completing agent is added. Calcium supplements were analysed using a method incorporating in-atomizer trapping in a graphite furnace. For a 1000-µl sample volume, the detection limit was 120 ng/l. This could be decreased to 30 ng/l if the ferricyanide was purified by cation exchange. Two materials from a local pharmacy were analysed, each of which contained 0.43 µg of lead per tablet. A procedure was then devised in which the lead was trapped on the interior of a slotted quartz tube in a fuel-lean flame. When 50 µl of MIBK was injected into the nebulizer, the lead was remobilized from the interior of the tube by the transient fuel-rich flame. The limit of detection was 28 ng/l for a 7.8-ml sample, and the procedure was applied to the determination of lead in NIST SRMs 2709 (San Joaquin soil: 19 mg/kg) and 1515 (apple leaves: 0.47 mg/kg). Finally, lead has been determined in wine by hydride generation following preconcentration by solid-phase extraction of the diethyldithiocarbamate complex on Chromsorb 102. Some efforts have been devoted to the optimization of the manifold design and operation, resulting in improvements in the sensitivity. Several designs of gas–liquid separator have been constructed and evaluated (though the original Perkin-Elmer design is possibly the best), and several ways of decreasing the contamination of the reagents, especially the ferricyanide, have been evaluated.

### Automation of electrochemical techniques

*H. G. Jayaratna, M. A. Ranalletta and C. S. Bruntlett, Bioanalytical Systems, 2701 Kent Avenue, West Lafayette, IN 47906, USA*

A flow-cell was developed for automating cyclic voltammetry (CV), stripping voltammetry (SV), stripping potentiometry (SP) and other conventional electrochemical techniques. The present design is made of polycarbonate material and allows the use of standard Ag/AgCl reference and solid electrodes, e.g. glassy carbon (GC) and gold (Au). The cell volume is ~70 µl.

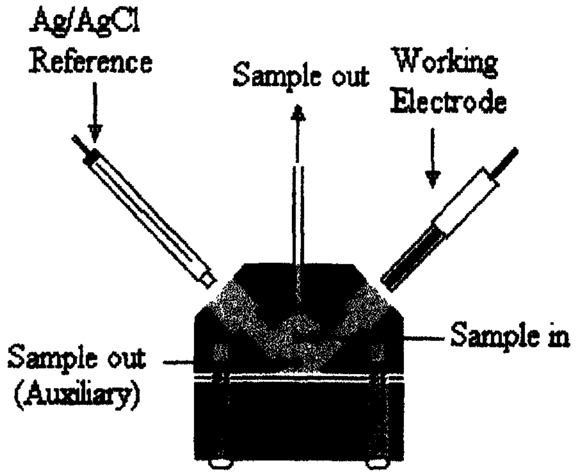

*Flow-cell design for conventional solid voltammetric electrodes. The holes for sample in and out in the middle of the cell cavity were placed on opposite sides. The top outlet served as the exit for air bubbles.*

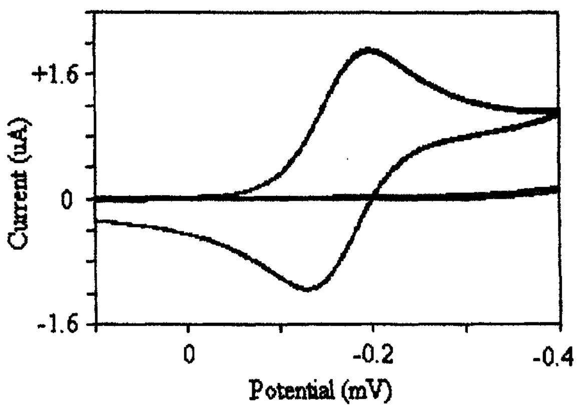

*An overlap of 15 CVs of $Ru(NH_3)_6Cl_3$ and 15 CVs of blank solution (KCl) collected from the automated system using a 1 mm GC electrode. E(ini) = +100 mV; E(fin) = −400 mV; Scan rate = 100 mV/sec. It can be seen that effective flushing of the cell cavity between samples was achieved with no carry-over effect. The flow rate used was 0.4 ml min$^{-1}$.*

Complete automation was achieved with a 30-vial auto-sampler. A peristaltic pump was used to deliver samples to the cell. It was then tested for flow profile and the ability to reproduce electrochemical data. Sample throughput depended on the technique, the concentration of the sample (deposition time) in the case of SV and the effectiveness of cell flushing.

Data from CV and SV (at mercury film electrodes) will be presented in this paper.

### Monitoring natural gas composition using infrared analysis

*Tye Ed Barber, Norma L. Ayala, John M. E. Storey[1] and Jeffrey S. Armfield[1], Department of Chemistry, Tennessee Technological University, Cookeville, TN 38505-5055, USA; [1]Oak Ridge National Laboratory, P.O. Box 2009, MS 8087, Oak Ridge, TN 37831-8087, USA*

Alternative transportation fuels have played an important role in domestic energy policy since the oil embargos of the 1970s. Besides offering energy security, there can be environmental benefits from using alternative fuels. Because of its widespread distribution system, natural gas has emerged as the most important near-term alternative to gasoline and diesel fuels. Currently, natural gas engines are used in a number of transportation applications. One major limitation to the increased usage of natural gas is the variable composition of natural gas. To minimize emissions and obtain maximum efficiency, natural gas engines are optimized to operate at certain air/fuel ratios and timing based on the combustion properties of a certain gas mixture. Because the composition of natural gas is variable, the combustion characteristics of the gas are also variable. This variation does not greatly influence the performance of simple burner systems, but it can dramatically impact the performance of engines. Engines burning fuel other than at the designed specifications can have higher emissions and shorter lifetimes. To address this problem, an infrared gas sensor is being developed that can determine natural gas composition. The goal of this work is to develop a sensor that can be installed on a vehicle allowing on-board determination of natural gas composition. Information from the sensor can be used to adjust engine parameters to correspond to the combustion characteristics of the natural gas. This will allow an engine to operate at maximum efficiency using a wide range of fuel compositions. In this paper, the sensor design and limitations of the sensor will be discussed.

### LIMS to instrument connectivity options: pros and cons

*Lisa Ventry Milenkovic and Mary Curtin Jackson*[1], *Quality Automation Sciences, 750 Verona Lake Dr., Weston, FL 33326, USA;* [1]*Massachusetts Water Resources Authority, Boston, MA 02129, USA*

One of the greatest improvements to overall data quality and laboratory efficiency achieved by a LIMS implementation is through interfacing of instruments to the LIMS. While most analytical instruments in the laboratory provide a standard RS232 serial I/O port, several methods exist to interface this port to the LIMS. In general, laboratories have taken four main approaches to interfacing.

(1) Through a data acquisition box.
(2) Direct to the LIMS through a terminal.
(3) Through an intermediate PC program.
(4) Via a network protocol file transfer.

In this paper, we present the pros and cons of these four different approaches using real-world examples of the interface of analytical balances to LIMS from different software vendors. We will also present a decision tree that can be used by the laboratory to extend this discussion to other analytical instrumentation and to determine the best method for their application.

### Web-based statistical quality control reporting

*J. Cawley, Northwest Analytical, 519 SW Park Ave., Portland, OR 97205, USA*

Web technology is an effective means to transmit laboratory SQC (statistical quality control) information to the laboratory's organization, its customers and regulators. The Web provides an alternative to the costs and technical limitations inherent in LIMS and manufacturing information systems when applied to enterprise-wide reporting of graphic information, e.g. control charts.

Conventional systems confront issues, e.g. mixed vendor network technology, multiple data sources, access problems, performance and scalability. Because SQC is graphics intensive, many legacy systems cannot cost effectively deliver control charts and process capability reports throughout the enterprise.

Web browsers provide the desirable alternate technology. The network exists, is independently supported, security is standardized, and browser-based systems are easier and less costly to scale. Web browsers provide a standard user interface that is optimized for high graphic content.

The design options for an effective SQC web server are discussed with a focus on web servers that provide SQC chart content to Web pages. Such a Web server can effectively provide the SQC reporting component for both LIMS and manufacturing information systems. A case study examines the system technical requirements and the results of using Web technology for LIMS-based SQC reporting.

### Personal computers have revolutionized laboratory information management systems (LIMS)

*Don Kolva, Accelerated Technology Laboratories, 496 Holly Grove School Road, West End, NC 27376, USA*

Personal Computers (PCs) have revolutionized the way that laboratories operate and has allowed them to manage their operation and increasing amounts of data. From sample login, to tracking samples though the lab, result entry, data analysis, QA/QC, and then reporting and invoicing, PCs have provided a means to effectively manage the laboratory information. Initially, lab notebooks were used to track this information, but once the chemistries became automated, the throughput increased and the early tracking systems were no longer sufficient.

The introduction of computers to the laboratory environment spawned the development of a number of proprietary LIMS, which allowed laboratories to keep up with the increased data from the automated analyses. These systems tended to be either mainframe- or mini-computer-based systems as PCs did not provide enough processor power to perform the complex operations. The computer hardware and maintenance costs of these early systems were often prohibitive for many small- to mid-sized laboratories.

In the 1990s, evolutions in PCs, networking and database engines converged to provide the necessary ingredients for PC-based LIMS. Networks were widely implemented and allowed many users to share information located on a server. PC-based database engines, e.g. Microsoft Access®, utilized this shared information. As PCs became more and more powerful, functional LIMS became a reality. The PC dramatically reduced the cost to implement a LIMS and provided the means to integrate the laboratory information with other applications running on the PC. This integration revolutionized the operation of the laboratory.

The next major technological advance in LIMS comes in sharing data over the intranet/internet via web browsers and a further increase in automation. This paper shall encompass the evolution of LIMS, with a focus on SQL databases, integrated CLP forms and electronic data deliverables. The laboratory of the future will also briefly be covered.

### Applications of computer-based training (CBT) for laboratory software

*Ronald Lee Hanson, LabSystems R&D, 1 St George's Court, Hanover Business Park, Altrincham, Cheshire WA14 5TP, UK*

The continuing growth of learning technology has seen many companies embracing the use of CBT materials. Provision of CBT materials by the software vendor gives a valuable aid in the support of new users and can significantly reduce the learning curve associated with laboratory software. CBT materials can be readily integrated into a company's existing computer-aided learning (CAL) system or be used as stand-alone instructional resource. This paper describes two applications.

(1) For end-user training in LIMS, the use of CBT materials provides a cheap and viable method for training large numbers of staff spread out over many geographic locations.
(2) Instrument and process simulations can be created to provide a demonstrable level of user competence while maintaining safety in the laboratory.

This paper will use examples created by LabSystems using Macromedia Authorware, although other applications will be discussed. It will also explain the role of CBT in the training of LIMS systems.

### Computer technology developing on-line manuals

*Stan D. Green, Applied Automation, Battlesville, OK 74006, USA*

Maintenance personnel can now sit at a workstation, remotely access any gas chromatograph in a plant and perform operational and maintenance functions that once could only be performed at the local analyser or in a lab. Likewise, support documentation for these tasks that was once contained in technical manuals now can reside as help files within a workstation. However, to be useful to maintenance personnel, these interactive help files must be designed not merely for aesthetics, but with the user in mind.

On-line documentation should be developed with an understanding of how a user will use it and what they will expect from it. For example, which information should be available as on-line tutorials, as opposed to context-sensitive help files, or what information should be structured for hard copy printouts. Likewise, access to the information must be quick and intuitive. Simple hypertrails should enable users to take different, yet structured, paths through the hypertext knowledge database dependent upon their particular tasks.

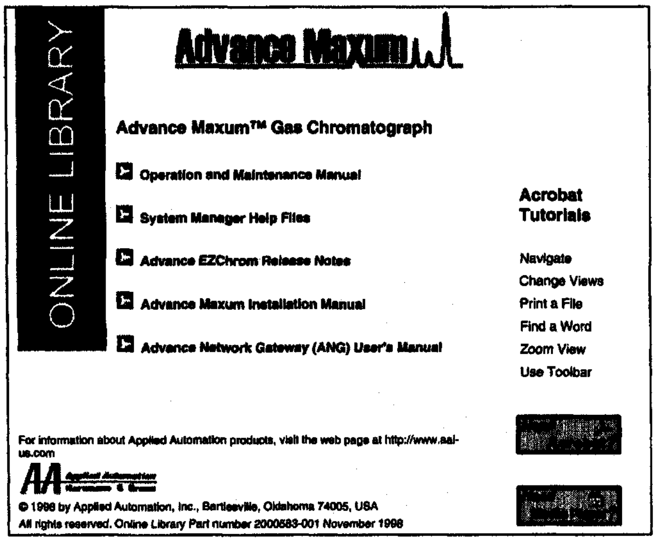

### Electronic lab notebook systems for R&D and testing labs: driving creation and acceptance for industry

*Rich Lysakowski, CENSA, 800 West Cummings Park, Suite 5400, Woburn, MA 01801, USA*

Collaborative electronic notebook systems (CENS) will eventually replace traditional paper-and-pen-based record-keeping systems with fully electronic, legally defensible, multimedia, multiuser systems that offer many advantages over paper. How soon will they be on the market? What's being done now to make them available to scientists and engineers? How will these record-keeping systems integrate with existing data management systems, e.g. LIMS, instrument data management, combinatorial chemistry and high-throughput screening applications? What about the various wireless, hand-held notebooks, PDAs and other portable devices—how will their ephemeral datasets be transported and secured in an emergent record-keeping infrastructure?

The Collaborative Electronic Notebook Systems Association (CENSA) is an international professional and trade association formed in late 1996 to answer these questions and many more. CENSA's mission is to drive creation and global acceptance of highly functional and usable electronic notebook systems that meet all legal, regulatory and scientific requirements. CENSA is hosting an international consortium of end user and vendor corporations called the CENS Consortium. These major corporations and government laboratories are now specifying, designing, developing and testing commercially supported collaborative electronic notebook system hardware and software. The consortium-created technologies will rapidly spill over into many other industries as the CENS products and standards make their way to the open market. Applications for CENS are many, and include product research, development, manufacturing and testing in high-throughput screening, analytical chemistry, biotechnology, healthcare, environmental and related areas.

This paper will cover the mission, objectives and progress of CENSA on its industrial research and product development objectives.

### Use of hand-held computers for remote sample collection, instrument integration and logistics tracking

*Kevin Peter Smith, LabSystems R&D, 1 St George's Court, Hanover Business Park, Altrincham, Cheshire WA14 5TP, UK*

The advent of powerful hand-held computers provides opportunities for greatly improved productivity and quality of data. This paper describes three novel applications.

(1) For field-based sample collection, the use of a hand-held PC to enter pertinent data (temperature, grid reference, etc) which can then be uploaded into a LIMS as part of the sample registration process.
(2) For collecting data for LIMS, chromatography or other data systems directly from instruments where the use of a regular PC is prohibited due to size, e.g. within fume cupboards, etc.

(3) For tracking samples and laboratory inventory, including disposition and freezer management.

This paper will use examples based on Microsoft Windows CE™-based devices, although products, e.g. the Psion and Palm Pilot will be discussed. It will also explain the use of infrared communications to ensure maximum mobility of the operator.

### A new technology for on-line information storage and retrieval in the scientific environment

*David M. Levy, Mantra Software, 1900 West Park Drive, Westborough, MA 01581, USA*

The role of the NuGenesis™ scientific data management system (SDMS) in the scientific environment is to capture and unify virtually all Windows-based information into a common database. This technology facilitates the ability of the scientist to focus more on science and less on figuring out how to unify, organize and utilize data from the numerous sources in the laboratory and corporate infrastructure.

NuGenesis SDMS is a combination of three technologies. First, a patent pending, 'Print to Database' technology which can take any Windows 95, 98 or Windows NT printed output and print the image of the report to an Oracle or Access database in a vector-scalable, text-searchable format. User-defined 'tag' information and system level 'tag' information is associated with the data being printed to the database. Second, is the database itself, which serves as an electronic on-line report repository for Windows-based analytical applications, e.g. chromatography, mass spectrometry and other analytical software, as well as business applications, e.g. Microsoft® Word and PowerPoint. Virtually anything that can be printed in the Windows environment can be printed to a NuGenesis SDMS database. Third, is a sophisticated viewer and extractor of information from the database which enables the user to search, export, review, print, archive and send information between the NuGenesis SDMS database and third party applications. This viewer has the ability to search the 'tag' information as well as being able to search information within the reports, which are inside the database.

NuGenesis SDMS technology provides a unique mechanism to rapidly consolidate data from multiple analytical and business applications into a single database architecture, providing an enterprise-scalable system for managing data, information and knowledge across the corporation. Furthermore, operations, e.g. creating compound documents, which are extremely difficult with existing technology, can now be done in minutes and re-archived to the NuGenesis SDMS database in order to make that information available to the enterprise.

### Workplace monitoring of carbonyls with modular derivatizing agents

*Christine Kempter and Uwe Karst, Westfälische Wilhelms-Universität Münster, Anorganisch-Chemisches Institut, Wilhelm-Klemm-Str. 8, D-48149 Münster, Germany*

Aldehydes and ketones are important volatile and reactive substances. Their widespread use in industries and their presence in the environment connected with their toxicological properties requires their quantitation. The determination of these substances in complex matrices succeeds with their derivatization and the subsequent separation of the corresponding derivatives by means of reversed-phase-HPLC. In this presentation, we will describe the synthesis and application of taylor-made agents for the precolumn derivatization.

The trifunctional cyanuric chloride suits for the stepwise synthesis of such a modular derivatizing agent. With increasing temperature, the three chlorine atoms are selectively replaced by a detectable moiety, a reactive moiety and a polarity-modifying moiety. The result of this procedure is a derivatizing agent with the desired reactivity, detectability and chromatographic properties. The introduction of a fluorescent group allows extremely low detection limits for many aldehydes.

A fluorescent modular derivatizing agent was synthesized. A suitable reactive group for the derivatization of carbonyl compounds is the hydrazine group. As fluorophor, a naphthyl group was linked to the molecule. An amino function, replacing the third chlorine atom in the molecule, modifies the polarity and protects the derivatizing agent from polymerization. Several derivatives of the carbonyls were synthesized, and their spectroscopic and chromatographic properties were investigated. Air samples were analysed and the results were compared to established hydrazine agents.

### Integrated process analysis

*M. P. Collins and R. M. Belchamber, Process Analysis and Automation, Fernhill Road, Farnborough, Hampshire GU14 9RX, UK*

In recent years, near-infrared spectroscopy has risen to the forefront in process analysis. Many of the advantages of NIR are also to be found in other regions of the spectrum, i.e. the UV and visible regions. Fibre optics can still be used to make measurements in hazardous areas. Many compounds including drug substances, anti-oxidants and aromatic solvents are strong UV absorbers. Chemometrics can be used to analyse multi-component mixtures.

Where UV absorbances are too strong to measure using normal transmission methods, ATR probes are used thereby extending the dynamic range of the measurement.

We have developed a number of successful applications using optical probes, photodiode array spectrophotometers and object-orientated software. These have included anti-oxidants in polyolefin melts, cleaning validation in the pharmaceutical industry, and the analysis of mixed solvent streams.

The link in these applications has been to use LabVIEW (National Instruments), a software development tool for instrumentation. We have developed a chemometric tool kit (Charm Works) for incorporation into LabVIEW applications.

We use a four-layer model for the software design. Layer 1 is the instrument driver (interface). Each instrument has a bespoke interface common to all applications of that instrument. Layer 2 is the spectroscopic functional layer where procedures, e.g. signal integration, dark signal correction and calculation of absorbance are collected together. Layer 3 is where chemometrics are performed. The final layer is the user and system interface. This is custom designed to permit the ultimate flexibility in systems integration.

### Recent developments in the design of atomic fluorescence spectrometers to allow arsenic speciation measurements on a routine basis

*W. T. Corns and P. B. Stockwell, PS Analytical, Arthur House, Crayfields Industrial Estate, Main Road, Orpington, Kent BR5 3HP, UK*

The toxicity and biochemical behaviour of arsenic species relies on their chemical forms.

Toxic inorganic arsenic species (arsenate and arsenite) are biomethylated by bacteria, fungi, algae, invertebrates and man to yield monomethylarsonic acid (MMA) and dimethylarsinic acid (DMA) which are considered less toxic than the inorganic forms. In this paper, a system utilizing strong anion exchange (SAX) ion chromatography coupled to hydride generation atomic fluorescence spectrometry will be described.

Non-reducible forms of arsenic, e.g. arsenobetaine and arsenochlorine, are determined using on-line UV photodegradation. Absolute limits of detection are found to be below 10 pg of arsenic for each species. A range of applications for environmental matrices will be presented.

### On-line analysis of liquid streams for mercury in the chloralkali industry—experiences of an installed instrument base

*W. T. Corns, P. B. Stockwell and N. K. Brahma, PS Analytical, Arthur House, Crayfields Industrial Estate, Main Road, Orpington, Kent BR5 3HP, UK*

Mercury has had and continues to have an important role in the chloralkali industry. Controlling and monitoring the analyte to achieve zero mercury emission levels poses distinctive challenges for the analytical chemist.

The authors have cooperated to develop a significant advance in methodology by applying discrete sample injection techniques to analyse samples, e.g. 50% caustic soda on a repetitive basis. Previously, this approach had shown useful applications for the analysis of mercury in 98% sulphuric acid. Such an approach offers advantages over conventional on-line measurements as very small samples are introduced to the instrumentation, thus avoiding matrix interference effects. The atomic fluorescence detection system, with its low detection levels and wide linear dynamic range, allows this procedure to be a viable option in the chloralkali industry. Instrumental systems have been developed for the analysis of mercury in 50% caustic streams and associated effluent streams.

The challenges provided by these measurements and the procedures developed to analyse the mercury levels routinely and automatically will be described in this paper. Results will be presented from an installed monitoring system.

### Sample Wizard: a unique automatic program for sample preparation, calculation and documentation

*Blaha Dave, Zang Li-Hsin, Deguchi Kisaburo and Safferthal Rudy*[1]*, Hitachi Instruments, San Jose, CA 95134, USA;* [1]*Merck KgaA, SLP Fo BS, 64271 Darmstadt, Germany*

We have developed unique Sample Wizard software for sample preparation, calculation and documentation. This program can help users to design a sample preparation (dilution) procedure graphically, which can then be saved.

The concentrations for all intermediate and final solutions are calculated automatically. Backward calculation is also available so that the amount of solute or amount of solvent will be calculated if a desired concentration is entered. With the graphic display, it is easy for operators to prepare samples by following a procedure. A report can also be printed to show the actual measurements at each step when a procedure is followed. Sample weights and volumes can also be imported into Sample Wizard from a file and used in a procedure. The complete sample information including component names and concentrations can be stored as sets of samples.

The information can then be used as input in sample analysis. Currently, Hitachi's D7000 HPLC data station can use the component and concentration information stored with the samples to perform calibration and quantitative analysis if these samples were prepared using Sample Wizard.

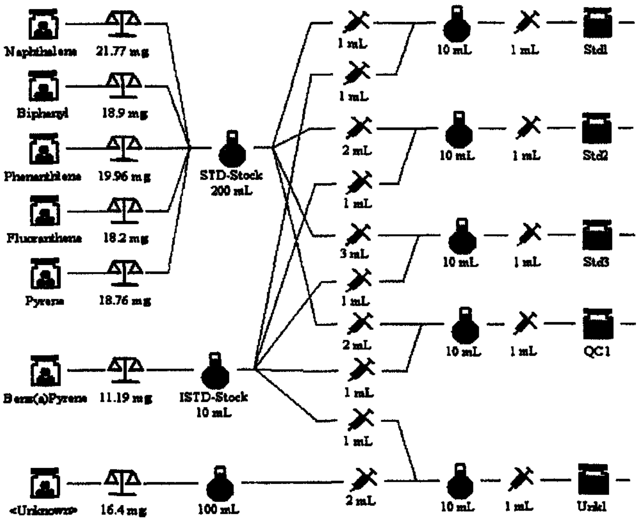

*Example sample wizard procedure.*

### Instrumental analysis taught using virtual analytical instrumentation

*Donald B. Nuzzio, Analytical Instrument Systems, P.O. Box 458, Flemington, NJ 08822-0458, USA*

The purpose of this paper is to illustrate how an instrumental analysis course can be taught using virtual instrumentation. Basic principles in separation science, spectroscopy and electrochemistry can all be simulated on a computer. By simulating analytical instrumentation on the computer, students can learn how to interpret data from real analytical instruments without running the instrument themselves. This method of instruction frees up valuable lab time and allows all students to interact with data from instrumentation when the department may not have or has limited access time for each student.

The next level of using virtual analytical instrumentation is in the actual control of instruments in the laboratory. Typically the analogue portion of the experiment can be made into a basic module and that in turn connected to a computer for control via a standard interface card. This allows the instructor the flexibility required in an instrumental analysis course by allowing him/her to configure lab modules to fit the number of students in the course.

Using this approach, we can now expose the student to state-of-the-art analytical instrumentation for the best possible education. Thus, the cost savings using virtual analytical instruments allows the student to be instructed on state-of-the-art equipment with an associated low cost to the university.

The following analytical instrumentation has been constructed using a minimum 486-66 MHz computer running Windows 3.1, 3.11 or Windows 95. The system includes the use of Labview software as the user interface, a National Instruments data acquisition board, and a dedicated analogue front end designed by Analytical Instrument Systems. The beauty of such a system is that the computer interface (hardware) does not have to be changed with the use of various analytical instruments. One simply disconnects the cable from one instrument and plugs it into another and runs a different software driver.

The following analytical instruments can be seen in this paper, the AIS Model DLK-1000, visible spectrophotometer, wavelength range from 330 to 850 nm, the AIS Model DLK-50/60, electrochemical analyser, and the AIS Model LCC-100, four-channel integrator for chromatography.

### Description of a new multipoint water sampler for continuous, on-line sampling and analysis of VOCs by purge-and-trap gas chromatography

*Michael L. Duffy and Nate Rawls, OI Analytical, P.O. Box 9010, College Station, TX 77842-9010, USA*

Laboratory analysis of water samples for volatile organic compounds (VOCS) by purge-and-trap GC and GC/MS has been performed for over 30 years. Methods for performing this analysis have been clearly defined by USEPA and other regulatory authorities. USEPA methods, e.g. 8020, 8240, 502.2, 601, 602, 624 and 525 all clearly specify defined laboratory techniques for low-level (ppb) analysis of VOCs in water.

When trying to apply these methods, with their strictly defined sampling protocols, to continuous on-line analysis, a compromise is typically required. The resulting procedure often involves the use of manual collection of water samples in 40-ml VOA vials and subsequent automation of the analysis on these collected vials using a laboratory-based purge-and-trap autosampler.

This paper describes a new multipoint autosampler that permits automated, sequential sampling of pressurized process water streams, thus avoiding the need for any manual collection of samples. The system described allows continuous, 24 h, automated sampling following USEPA-specified sampling requirements. Continuous sampling of up to six separate water streams, in any operator-defined sequential order, is possible with this system. System rinses and analysis of water blanks is also operator programmable. System specifications and descriptions, as well as results obtained on purge-and-trap GC systems configured according to USEPA methodology will be reported.

### Design, development and testing of a microprocessor-controlled automated short-path thermal desorption apparatus

*Vinod T. Das, John J. Manura[1], Chris Baker[1], Thomas G. Hartman, John Manos[1], Roland Roadenbaugh[1], Dan Lieske[1] and John Miller[1], Department of Food Science, Rutgers University, New Brunswick, NJ 08903, USA; [1]Scientific Instrument Services, Ringoes, NJ 08816, USA*

An automated, microprocessor-controlled thermal desorption unit GC accessory has been designed and built. The apparatus has been tested for mechanical ruggedness, baseline blanks, repeatability and efficiency. Mechanical performance was flawless, and a clean sample blank was obtained under high sensitivity GC-MS conditions. The system operates by user-defined parameters from custom-developed software with a graphical user interface (GUI). Samples of *n*-paraffin standards were analysed in series to determine repeatability. The RSD (relative standard deviation) of these samples using tetradecane and hexadecane as an indicator were 3.19% and 2.79%, respectively.

Further data which will be presented in this paper are calibration curves of reference standards, precision by direct thermal desorption, and purge-and-trap thermal desorption. Illustration on next page.

### How to use an electronic nose for on-line monitoring

*Wolf Muenchmeyer and Andreas Walte, WMA Airsense Analysentechnik GmbH, Hagenowerstr. 73, D-19061 Schwerin, Germany*

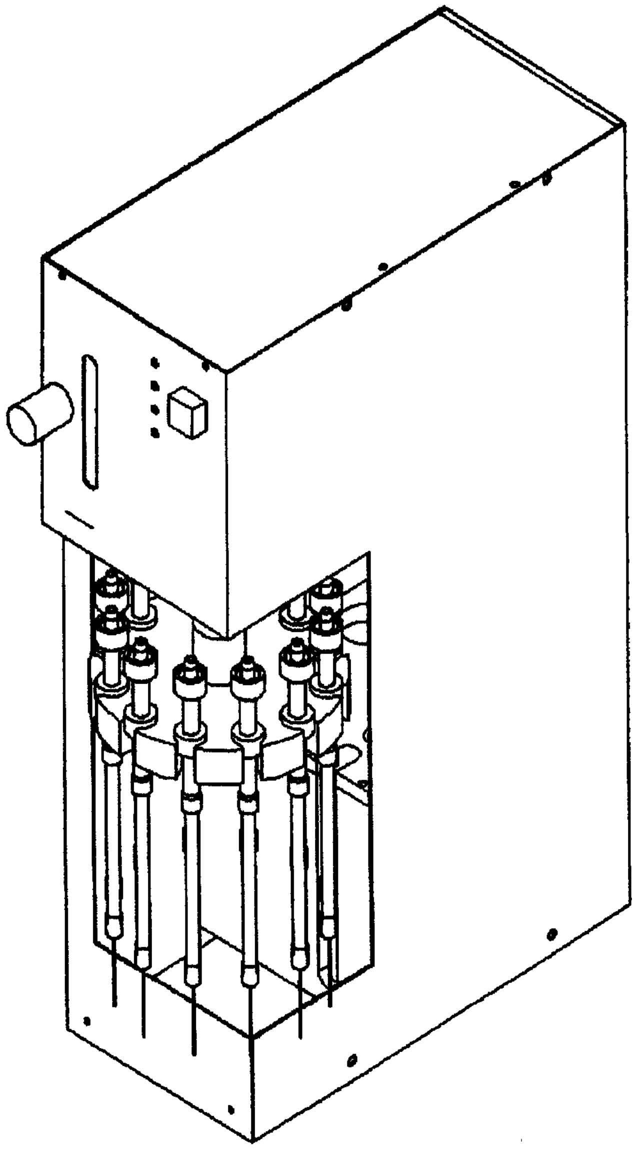

Electronic noses usually consist of an array of simple sensors, e.g. metal oxide semiconductors (MOS), conductive polymers (CP) or coated oscillators, e.g. quartz crystal microbalances (QCM) or surface acoustic wave oscillators (SAW) combined with the required electronics and chemometrical software. One of the main drawbacks of the sensors is their response to non-interesting ambient factors, e.g. temperature, pressure and humidity. For on-line monitoring applications, we have chosen MOS sensors because of their lower humidity response in typical ambient air applications and their low detection limits for organic compounds.

The MOS and CP sensors are non-linear sensors, which implies a concentration-dependent pattern of samples. For many on-line applications, it is necessary to have a recognition of the sample with no dependency of the concentration. The sampling method for achieving concentration-independent patterns will be shown in this paper.

Another problem of the sensors is the drift of the sensor signal with time. Using a zero-gas and differential measuring technique, the possible drift of the sensor signal is reduced. With a span-gas from an internal permeation device, the sensor response can be controlled.

With an optional enrichment unit, the detection limit for organic compounds is decreased into the low ppb range and the selectivity of the electronic nose can be adapted by choosing the appropriate adsorbent.

### Continuous monitoring of trout farm effluent before and after composting waste treatment

*Karen D. Bradham and J. Roger Bacon, Department of Chemistry and Physics, Western Carolina University, Cullowhee, N.C. 28723, USA*

The southern Appalachian Mountains of western North Carolina are blessed with an abundance of cold-water mountain streams that have been profitably exploited for trout aquaculture over the past several decades. Trout aquaculture represents one of the few agricultural pursuits which remains of economic importance to this region, and is in a position to significantly increase production levels. Effluent from trout farms, however, can negatively influence the quality of the water in streams along which they are located. The waste products produced during trout rearing originate directly or indirectly from the diets fed to the fish, and are composed of suspended and settable organic solids (fish faeces and uneaten feed) and dissolved nutrients (principally nitrogen and phosphorus). Excessive increases in levels of nitrogen- and phosphorous-bearing compounds originating from trout farm effluent could lead to eutrophication of the streams. Attempts were made to continuously monitor temperature, conductivity, pH, and ammonium and nitrate ion concentrations using Vernier™ probes. The probes were attached to a Blue Earth™ single-board computer programmed in BASIC for customized data collection, and were solar and battery powered to operate for weeks at a time collecting experimental data. Difficulties in continuous monitoring were encountered and will be discussed in this paper. Inorganic phosphorus was measured by malachite green calorimetric techniques. The data obtained are analysed in relation to per unit ton of fish feed operating with an alternative waste management treatment, a composting solid waste removal system. The data obtained indicate the magnitude of release of nitrogen and phosphate in the effluent from a commercial trout farm when operating with a composting waste treatment system.

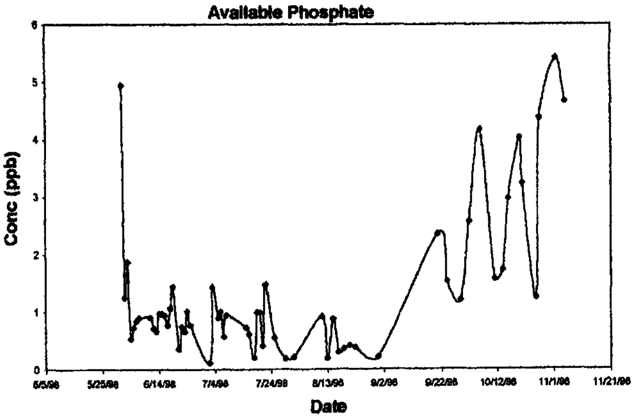

### Comparison between olfactometry and a portable nose for the evaluation of environmental odours

*Wolf Muenchmeyer, Andreas Walte and Daniela Jakobs*[1], *WMA Airsense Analysentechnik GmbH, Hagenowerstr. 73, D-19061 Schwerin, Germany;* [1]*RWTH Aachen, ISA, D-52074 Aachen, Germany*

The evaluation of bad odours in the environment is performed by olfactometry, which usually requires an expert team of at least four trained human noses. The environmental industry often needs a cheaper tool for monitoring the odours.

A new small portable electronic nose equipped with 10 metal oxide sensors was used for measuring and recognizing odour at gas filters in different stages of a sewage purification plant. The internal pumps of the electronic nose were used for the direct introduction of the ambient air and gases from sampling bags required for the olfactometry.

The electronic nose is capable of distinguishing between the gases in the different stages of the plant. A good correlation was possible in the analysis of the unfiltered gas and the gases after the first filtration units. The evaluation of the filtered gas at the end of the biological filtration system, in terms of human odour units, requires a higher selectivity of the system. The approach using an optional selective-enrichment unit is currently under investigation.

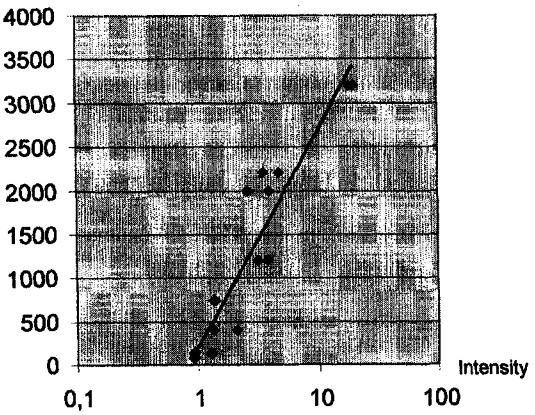

*Correlation between olfactometry and the portable electronic nose PEN at a sewage purification plant.*

### Automatic enrichment device unit for electronic noses

*Wolf Muenchmeyer and Andreas Walte, WMA Airsense Analysentechnik GmbH, Hagenowerstr. 73, D-1 9061 Schwerin, Germany*

Electronic noses usually consist of an array of simple sensors, e.g. metal oxide semiconductors, conductive polymers or coated oscillators, e.g. quartz crystal microbalances or surface acoustic wave oscillators combined with the required electronics and chemometrical software. In some applications, the detection limit of ~1–10 ppm for the semiconductors and 10–100 ppm for the oscillators is too high. Sometimes there is also a higher selectivity needed.

With an external enrichment unit, it is possible to lower the detection limit by one or two dimensions, depending on the sampling time and the retention capabilities of the adsorptive material. By choosing an optimal adsorptive material with the corresponding sampling temperature, also a selective enrichment is possible. Cross-sensitivities to humidity and very volatile compounds or permanent gases are minimized. After sampling, the adsorption tubes are thermally desorbed and analysed. Applications are, e.g. the analysis of aroma in alcoholic beverages, the analysis of odours in environments with high methane concentrations or environments with changing permanent gas concentrations.

The new small enrichment unit was used in combination with a portable electronic nose. Different applications using the enrichment unit with an automatic sampling, thermal desorption and consecutive analysis are shown in this paper.

### Automatic solid phase injector (ASP) or how to introduce any sample in a GC or GC/MS without extraction

*Philippe Mottay and Philippe Parsy*[1], *Mass Evolution, 330 Meadowfern Dr., Suite #113, Houston, TX 77067, USA;* [1]*I.D. Analytical Services, 14 Blvd Anatole, F-86000 Poitiers, France*

Direct introduction in mass spectrometry is well known, but does not offer the capabilities of separation of the GC. The new-patented (US Patent #5,714,677) ASP injector combines the MS solid probe and GC separation. It is now possible to inject all matrices directly in a GC without extraction.

Liquids or solids are introduced with different mobile units into the ASP injector, manually or with an automatic liquid sampler.

The filament mobile unit allows evaporation of the solvent (including water), fast derivatization under inert atmosphere on the same sample and elimination of non-volatile residues after analysis. With this module, the

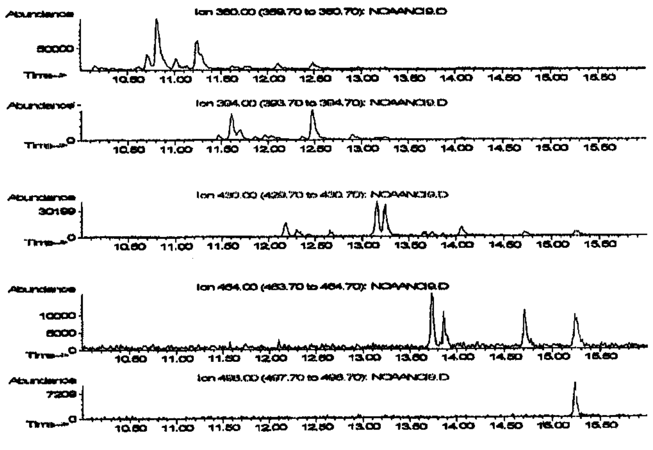

*Detection of PCBs in a soil sample directly desorbed.*

pyrolysis of polymers is performed on the same sample after analyses of volatile compounds.

Other mobile units allow introduction of solids, e.g. fibres (hair, wood, textiles, metallic wires, etc.) or powder samples (soil, wood, leaves, tobacco, coffee, washing powder, etc.).

The mobile units, the ASP uniqueness, can be exchanged allowing numerous injection possibilities in many analytical areas.

Some results in pharmaceuticals, cosmetics, forensics, foods, pesticides and petrochemicals are presented in this paper.

In GC/MS, the ASP injector combines the friendliness of mass spectrometry direct introduction probe with the selectivity of gas chromatography.

### Speciation of mercury levels in natural gas condensates by atomic fluorescence

*W. T. Corns, P. B. Stockwell, Azman Bin Shafawi[1], M. Foulkes[1] and L. Ebdon[1], PS Analytical, Arthur House, Crayfields Industrial Estate, Main Road, Orpington, Kent BR5 3HP, UK; [1]Department of Environmental Science, University of Plymouth, Drake Circus, Plymouth, Devon PL4 8AA, UK*

Mercury levels and the distribution of the various species of mercury in natural gas condensates are of vital importance in the petrochemical industry. Armed with details of the total level of mercury in these condensates, the petrochemist can gain an insight into the extent of the problem. However, the design of mercury removal strategies requires an accurate and reliable method to define the mercury species profile or fingerprint. A simple and effective method to obtain this profile will be described in this paper.

The instrumentation utilizes a PTV injection system, a gas chromatography oven with temperature programming capability and an atomic fluorescence detector coupled to the GC column via a pyrolysis oven. Samples of the condensates are introduced directly at the injection point and the mercury compounds are separated according to a simple temperature programme routine.

Results obtained from a range of condensates will be shown, illustrating the capabilities of this approach. It will be shown that this approach provides vital information to check or evaluate mercury removal strategies. Results obtained from condensates where mercury has been claimed to be removed and the reason why this has not occurred will be presented.

### Development of an on-line measurement system for a high degree of dew point

*Kijang Oh, Dalwoo Kim, Sungwook Jung and Choongsoo Lim, Instrumentation Research Team, RIST, #32 Hyosa-Dong, Pohang, Kyungpook 790-330, Korea*

We have developed a continuous measurement system of high dew point (40–70 °C) of the atmospheric gas (above 850 °C) in a continuous annealing line (CAL). The control of the dew point in the CAL is essential for the improvement of the quality of electrical steel.

We found an empirical formula between dew point and the amount of water in the atmospheric gas, and applied the formula at the dew point measurement apparatus. The main part of the apparatus is a gas chromatograph which is used to measure the amount of water in the dew point continuously using gas chromatography and is transformed into the dew point with a data processing system. The measurement range of this apparatus is 10–80 °C. In order to prevent the condensation of vapour between the apparatus and a furnace, we heated up the connection pipe with a heating band.

By comparing the measurement results with those of conventional methods, we found that our new type of measurement system is reliable for the on-line measurement of high dew point in the production line.

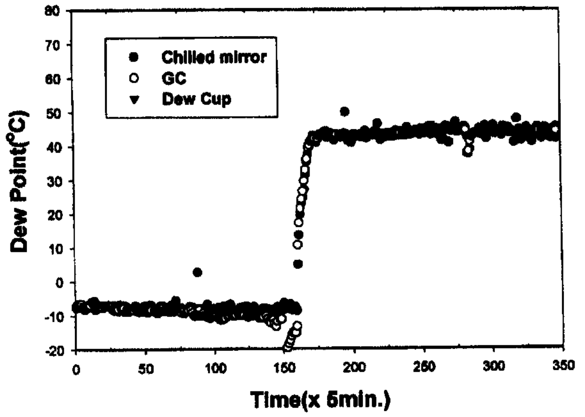

*Results of dew point using GC compared with those of conventional methods in a production line.*

### Automated determination of organic lab air toxics by thermal desorption

*Joseph W. Walsh, Scott E. Adams, Alltech Associates, 2701 Carolean Industrial Drive, State College, PA 16801, USA*

A thermal desorption method has been developed for the determination of organic lab air toxics. The method allows automated unattended determinations. A vacuum pump pulls air samples into the sorbent tube of a thermal desorption unit. Once the analytes are trapped by the adsorbent tube, residual air and moisture is purged from the tube by carrier gas flowing in the same direction that the sampling took place. The carrier gas direction is then reversed and the tube is heated. This desorbs analytes to a secondary focusing trap which reconcentrates the analytes from the sampling trap. Secondary focusing eliminates the need for cryofocusing otherwise required when desorbing large-capacity sampling traps. Analytes are rapidly transferred from the high-capacity sampling trap to the focusing trap under high heat and flow conditions. The focusing trap is then backflushed while heating under capillary compatible flow rates which transfers the analytes to the GC system for separation and identification. The thermal desorption system controls the sampling and recycling times, and communicates to the GC to determine ready status and to initiate the GC run. Details of the sampling protocols and the chromatographic conditions will be shown in this paper.

### A new software program for optimization and post-run data processing of the GC analyses on a dual-gated pulsed flame photometric detector (PFPD)

*Michael, L. Duffy, Richard Simon, and Ronald Snelling, OI Analytical, P.O. Box 9010, College Station, TX 77842-9010, USA*

The pulsed flame photometric detector (PFPD) is a sensitive and selective GC detector used in the analysis of various heteroatoms, particularly sulphur and phosphorus. This paper will introduce a new software package that provides an easy-to-use, post-run optimization and data analysis of the PFPD's dual-gate models of operation.

The software permits post-run viewing of every pulse's chemiluminescence signal, which can then be used to obtain structural information on unknown compounds encountered in the chromatographic analysis. Additionally, gate optimization on previously obtained chromatographic analyses and reprocessing of this PFPD data (in AIA formats) permits reintegration and quantitation by the GC's data system, based on the new gate parameters. Practical applications of these techniques will be presented with several applications involving the analysis of sulphur-, phosphorus- and nitrogen-containing compounds.

### Automated sample fractionation and analysis using a modular LC-GC system

*Frank David, Pat Sandra[1] and Andreas Hoffmann[2], Research Institute for Chromatography, Kennedypark 20, 8500 Kortrijk, Belgium; [1]University of Gent, Department of Organic Chemistry, Krijgslaan 281 S4, 9000 Gent, Belgium; [2]Gerstel GmbH, Aktienstraße 232–234, 45473 Mülheim an der Ruhr, Germany*

Conventional sample preparation methods used to isolate a fraction of interest from a complex matrix are mostly based on low-resolution techniques, e.g. liquid–liquid extraction, column chromatography (including gel permeation chromatography on Bio-Beads) or solid-phase extraction.

In comparison to these techniques, sample fractionation by HPLC offers a far superior efficiency and selectivity. Due to the higher resolving power, fractions are more specific, better defined, and the fractionation is more reproducible. HPLC fractionation can be automated and allows miniaturization, resulting in lower solvent consumption.

The combination of liquid chromatography with gas chromatography offers a highly selective sample preparation step that is integrated in the analytical system. LC-GC coupling has been used for several years and dedicated instrumentation has become available. Recently, a new, fully automated and flexible on-line LC-GC system was introduced. The system is based on standard LC and capillary GC instrumentation. The interface consists of a flow cell, a large volume sampler and a PTV inlet. Transfer of LC fractions from normal-phase LC, reversed-phase LC or size exclusion chromatography can be performed totally or partially. The LC fractions (usually 0.1–1 ml) are introduced in a PTV inlet in the solvent vent mode. After evacuation of the solvent, the inlet is programmed and the solutes are injected in the GC column. The whole process, including the LC fractionation, transfer, large volume injection and GC (or GC-MS) is controlled from a single PC station and is fully automated.

In this paper, the applicability of the on-line LC-GC system will be demonstrated for the determination of pesticides in essential oils, the fractionation of petrochemicals and the determination of PCBs in oil and fat matrices.

### Monitoring the multisulphonated zinc phthalocyanine synthesis reaction by capillary electrophoresis with LIF detection

*Marcia R. Santana, Yuzuru De Abreu, Marina F. M. Tavares and Joel C. Rubim[1], Instituto de Química, Universidade de São Paulo, C. P. 26077, 05599-970, São Paulo, Brazil; [1]Universidade de Brasília, C.P. 04478, 70919-970, Brasília, D F, Brazil*

The photodynamic therapy (PDT) of cancer is a non-invasive technique that uses photosensitizing drugs, which upon illumination by an appropriate light wavelength, generate cytotoxic species, e.g. singlet oxygen. Multisulphonated zinc phthalocyanines (ZnPcS) represent a second-generation of drugs for PDT, with remarkable performance in clinical trials.

In this work, the synthesis of ZnPcS by the condensation method was evaluated as a function of time. The procedure employs equal amounts of ftalic and 4-sulphoftalic acids in the presence of a large excess of urea and catalyst. Aliquots of the reactional medium were extracted continuously and monitored by a CE-LIF equipment (P/ACE 5100, Beckman Instruments, semiconductor laser source, $\lambda_{exc} = 635$ nm, $\lambda_{ems} = 675$ nm).

An important variable during optimization of the analysis conditions was the conditioning of the capillary. By consecutive rinses of dil. $HNO_3$ (3 min), $H_2O$ (6 min), NaOH (3 min), $H_2O$ (6 min) and buffer solution (20 min), a better reproducibility of migration times

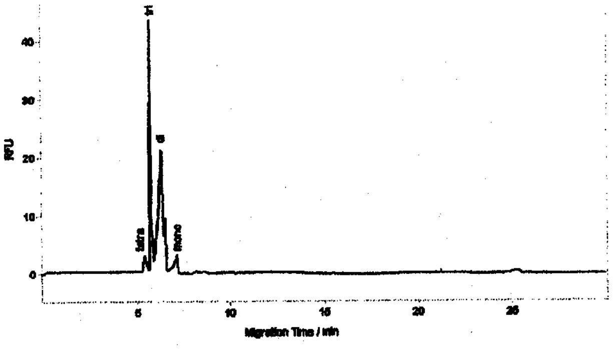

*Electroferogram of an aliquot taken at 53 min of reaction.*

was obtained. The best peak resolution for inspection of positional isomers of each sulphonated species was achieved when high buffer concentrations and surfactant were used (100 mM citrate buffer solution, pH 2.5 and 100 mM SDS). The formation of multisulphonated species was observed after 23 min from the mixing of reactants. The identification of the observed peaks was achieved by means of commercially available standards (porphyrin prod.). Peak area and height were used to follow up the production of multisulphonated species during the synthesis. The results revealed a distinct predominancy of multisulphonated species throughout the reaction time. A large amount of trisulphonated species followed by disulphonated, mono- and tetrasulphonated species was found. These latter two species accounted for less than 10% of the total amount.

### New developments in high-throughput screening (HTS) for combinatorial chemistry

*Mark Capparella and J. Robert Bickler, YMC, 3233 Burnt Mill Drive, Wilmington, NC 28403, USA*

The utilization of HPLC to separate the plethora of compounds generated through combinatorial synthesis continues to grow each year. Current technology allows for the screening of ~200 samples per day per HPLC instrument. As the numbers of libraries increases so does the demand for more analyses. With instrumentation already running at maximum capacity, the only ways to increase throughput are to install new instrumentation or to run faster screens.

The current paradigm for HTS is a 4.6 × 50 mm ODS column operated at 3–4 ml/min flows and 3–4 min gradients (7–10 min cycle times) yielding ~200 samples/day/instrument. In this paper, we will show how these increased HTS demands have led to the development of new HPLC columns which increase productivity up to seven times over the current technology.

### A new high-capacity automatic microplate loader for an HPLC autosampler

*John Hodgin and Steve Hydo, Alcott Chromatography, 5680 Oakbrook Parkway, Suite 148, Norcross, GA 30093, USA*

Combinatorial techniques have provided researchers with a powerful tool for scouting potential new drug discoveries. These techniques have also been expanded for applications in other areas of chemical product development. Combinatorial techniques generate thousands of compounds, or libraries, of potentially useful substances. A recent LC/GC magazine article described the characterization of a thiazolidinone library which contained 42 875 compounds. Large numbers of compounds such as these provide an interesting challenge for the analytical chemist responsible for the analysis of so many samples in a reasonable amount of time. The sample container most widely used for combinatorial methods is the 96-well microplate, and several analytical devices have been developed around 'microplate' technology. HPLC is not an exception.

The purpose of this paper is to describe the design and performance of a new automated microplate loader for the Alcott Model 718AL Autosampler. The loader, called the SPC 31, can hold up to 31 standard microplates or 13 'deep-well' microplates. The loader can handle either the 96-well or the 384-well microplate. In addition, this paper will describe a few applications of the device.

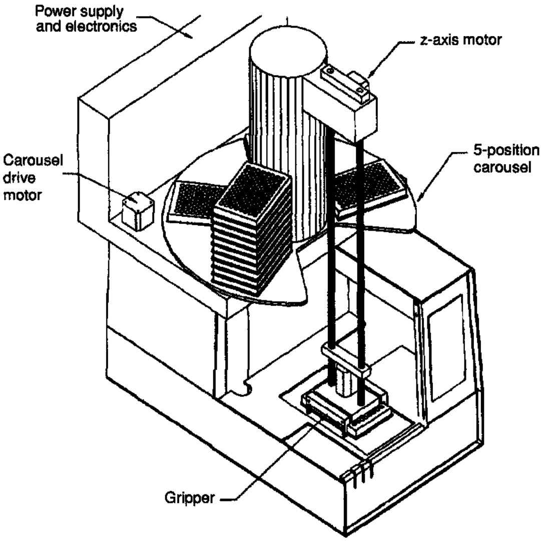

*Microplate loader.*

### An automated system for the purification of reaction mixtures using pre-packed disposable cartridges

*P. R. Regan, P. Rundle, T. Brewer, M. Cleeve and D. Ebdon, Jones Chromatography, New Road, Hengoed, Mid-Glamorgan CF82 8AU, UK*

A system is described for the automation of organic reaction mixture purification. The system makes use of a novel mechanism for the use of pre-packed disposable cartridges on-line, and includes a PC-based data system for system control and sample tracking. Data are presented which show the reproducibility of the system.

### Analysis of combinatorial libraries using HPLC and evaporative light scattering detection

*Melissa J. Wilcox and Michelle R. Chudy, Alltech Associates, 2051 Waukegan Road, Deerfield, IL 60015, USA*

Accelerated drug development has put increasing demands on pharmaceutical researchers to rapidly identify compounds created by combinatorial chemistry techniques. Many organic molecules, especially those of pharmaceutical interest, are relatively non-volatile, or thermally labile, or both, and many of these compounds are best separated by HPLC. Gradient reversed-phase HPLC is the separation method of choice for the majority of compounds created using combinatorial techniques.

Compounds generated through combinatorial synthesis are often analysed using HPLC in combination with low-wavelength UV and/or MS detection. While low-wavelength UV detection is a convenient qualitative method, it cannot be used for sample quantitation because UV absorption often bears no relationship to the relative masses represented by individual peaks in the chromatogram. Quantitation of combinatorial libraries by MS presents similar problems. Significant differences in ionization efficiency may exist between library components, meaning they would have to be quantitated based on the response of an appropriate series of external or internal standards. The considerable effort required to synthesize and characterize reference standards for each member of a compound library makes this approach impractical.

The evaporative light scattering detector (ELSD) eliminates the problems associated with low-wavelength UV and MS detection. Any sample less volatile than the mobile phase can be detected, regardless of its optical characteristics or ionization properties. Response is based on the sample's mass so that unknown components can be quantified by comparison to internal standards.

This work demonstrates the use of HPLC coupled with evaporative light scattering detection for the analysis and quantitation of pharmaceutical compounds. The advantages of evaporative light scattering detection over other types of HPLC detection will be discussed in this paper.

### Application of surface area and porosimetry analysis as a way to monitor the reproducibility of bonded silica gel batches in HPLC

*Ismail Rustamov, Faizy Ahmed and Toshihiko Hanai*[1]*, Phenomenex, 2320 W. 205th Street, Torrance, CA 90501, USA;* [1]*Health Research Foundation, Institute Pasteur 5F, Sakyo-Ku, Kyoto 606, Japan*

Reproducibility of the HPLC packing batches is one of the major concerns for chromatographers. HPLC column manufacturers have developed and implemented various tests which characterize different aspects of raw as well as bonded silica. Analysis of the results obtained from these tests helps not only to monitor the quality but also to improve reproducibility of the bonded batches.

Performance of the bonded material very much depends on the surface area, pore volume and pore diameter of the starting silica gel. Thus, the reproducibility of these parameters is a starting point for quality control of the final batches. At the same time, a particular batch of the silica gel may be used for bonding different batches of final material. For this reason, measuring surface and porosimetry values of the bonded phase will help not only to study changes in corresponding parameters, but also to monitor the quality and reproducibility factors.

This paper presents the results of extensive research of physiochemical values of the silica gels modified by organosilanes with different functional chains. Numerous tabular and graphic data illustrate the comparative results in an easy to understand manner. The paper also contains several batch-to-batch reproducibility charts. It shows that surface area and porosimetry analysis is a reliable way to monitor silica gels' bonding quality.

All physio-chemical analyses were conducted on Micromeretic's ASAP-2010 system by using BET and BJH calculation methods based on nitrogen adsorption and desorption isotherms.

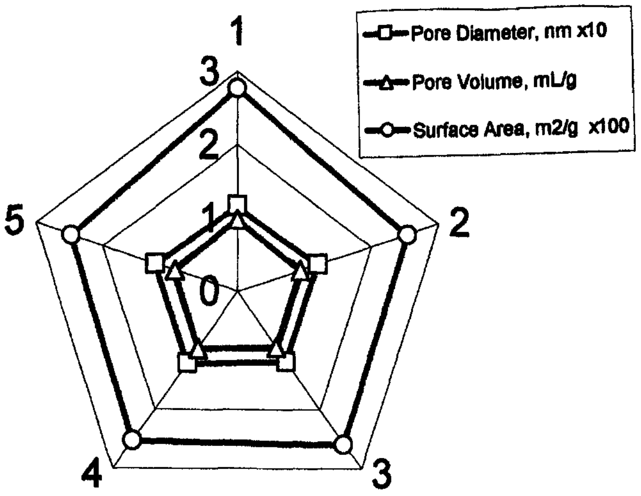

*Reproducibility of Jupiter 5C18 batches.*

### Development of digestion methods for the determination of total arsenic in a variety of foods

*Barbara S. Barnes, John R. Urban, Brenda S. Zimmer and Douglas T. Heitkemper, US Food and Drug Administration, Forensic Chemistry Center, 6751 Steger Drive, Cincinnati, OH 45237-3097, USA*

The determination of total arsenic and arsenic species in food is of importance for risk assessments. The FDA's Forensic Chemistry Center has a variety of crop samples including rice, wheat, carrots and potatoes that were collected in the early 1980s for a joint project between the FDA, EPA and USDA. These samples have been stored under nitrogen and frozen. Currently, these crop samples are being used in a joint FDA, EPA arsenic speciation project. Methods for the extraction of arsenic species from these samples are currently under development. In order to determine the efficiency and effectiveness of these extractions, the determination of total As content of the food needs to be determined. Microwave acid digestion, conventional acid digestion and dry ashing methods will be compared for efficiency and effectiveness in the preparation of these samples.

### Determination of ultra-trace amounts of cadmium, cobalt and nickel in sea-water by electrothermal atomic absorption spectrometry with on-line preconcentration

*Hui-Ju Chang, Yu-Hsiang Sung and Shang-Da Huang, Department of Chemistry, National Tsing Hua University, Hsinchu, Taiwan 300, Republic of China*

The direct determination of trace metals in sea-water by electrothermal atomic absorption spectrometry (ETAAS) is difficult even with sophisticated background correction and chemical modification; not only because of the presence of many trace metals at concentrations near or below the detection limit, but also because the sea-water matrix may cause serious interference. Preconcentration procedure can solve the above two problems and allow easy determination. On-line preconcentration system are better than the off-line batch preconcentration method because the former are more efficient, reproducible and easily automated, with a low consumption of sample and reagent and a low risk of contamination.

Chelex-100 has two major drawbacks to its performance for on-line preconcentration. These are: (i) the chelating resin undergoes a drastic volume change from the $NH_4^+$ to $H^+$ form; and (ii) the resin has an affinity for alkali and alkaline earth element that causes matrix problem during atomic absorption spectrometry of trace metal and decreases the resin's capacity. Muromac A-1 and Chelex-100 both contain iminodiacetic acid [-$CH_2$-$N(CH_2COOH)_2$] functional groups, but the Muromac A-1 chelating resin is better purified and does not swell or shrink.

In this work, a miniature column packed with Muromac A-1 chelating resin and a laboratory-built automatic on-line preconcentration system for electrothermal atomic absorption spectrometry were used to determine Cd, Co and Ni in sea-water. The preconcentration system was modified from a Perkin-Elmer AS-40 autosampler by mounting a Muromac A-1 microcolumn near the tip of the autosampler capillary, and we replaced the pull-and-push pump of the autosampler with a peristaltic pump. Retention of the metal ions as a complex on the micro-column was achieved by using Muromac A-1 as the chelating resin; 20% $HNO_3$ was then used for elution. The procedures of preconcentration were performed by using a four-way distribution valve and a programmable controller. Detection limits (and sample volumes) are $1.2 \times 10^{-4}$ μg/l for Cd (400 μl), 0.007 μg/l for Co (1800 μl) and 0.033 μg/l for Ni (800 μl). Relative standard deviations for the determination of Cd, Co and Ni in sea-water (CASS-3) were 2.9, 5.6 and 4.1%, respectively. The accuracy of the method was confirmed by the analysis of certified reference saline waters (NASS-4, CASS-3 and SLEW-1).

### Determination of Hg and Se in South San Francisco Bay water by atomic absorption spectroscopy with analyte concentration techniques

*Robert F. Wandro, Joe Theisen and Kenneth Lee, City of San Jose, Environmental Services Department, 4245 Zanker Road, San Jose, CA 95134, USA*

Both mercury and selenium have recently received considerable regulatory attention in San Francisco Bay. These elements can be toxic to birds and/or aquatic life, and bioconcentrate up the food chain. Hg and Se concentrations are among the properties the City of San Jose Environmental Services Department Laboratory has been monitoring at 12 sites in the South SF Bay on a biweekly basis for over 1 year.

A method for reliably determining Se concentrations at 0.10 ppb was developed and applied to estuarine samples from the South SF Bay. The technique of trapping the vapour from a hydride-forming element on the surface of an iridium-coated graphite tube (*J. Anal. At. Spectrom.*, **10** (1995), 1003) was applied to these samples. The system was semi-automated employing a Varian spectrometer system. Optimum deposition and atomization temperatures of 250 °C and 2000 °C, respectively, were utilized. A method detection limit of 0.002 ppb is calculated for digested samples following EPA protocol in 40 CFR Part 136.

Mercury determinations were performed using a modified EPA Method 1631. A method detection limit of 0.0008 ppb was achieved using a single gold trap and a Perkin Elmer FIMS atomic absorption system. South SF Bay sample concentrations typically varied between 0.010 ppb and 0.120 ppb.

A discussion of the methods and a summary of the results will be presented. In addition, the data quality will be evaluated using the results from triplicate samplings, equipment blanks, travel blanks, spiked samples and standard reference samples.

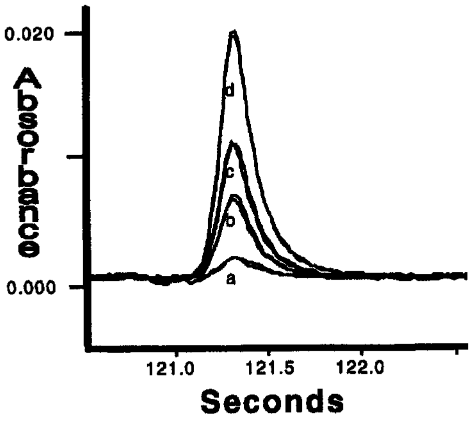

*Response-time curves in duplicate for selenium: (a) reagent blank, (b) 0.025 ppb, (c) 0.050 ppb, and (d) 0.100 ppb.*

### Sampling procedures for the determination of mercury in natural gas

*W. T. Corns and P. B. Stockwell, PS Analytical, Arthur House, Crayfields Industrial Estate, Main Road, Orpington, Kent BR5 3HP, UK*

There are several well-documented cases where mercury contamination in natural gas has resulted in costly plant shut-downs or even dangerous explosions. The mercury in natural gas can exist in several forms other than elemental mercury vapour. Natural gas sources from South East Asia are particularly prone to containing major levels of organomercury compounds. The levels of mercury are therefore a major cause of concern, posing difficulties in analysis and also problems for removal of the mercury prior to distribution and use.

Atomic fluorescence, in association with a purge-and-trap approach on gold-impregnated material, e.g. Amasil$^{TM}$, provides a well-accepted method for analysis of mercury at low levels in both on-line and off-line situations. For the latter approach, a suitable sampling procedure is necessary in order to obtain reliable and representative measurements, especially when the sampling source is at high pressure. A new sampling system will be described which allows samples of gas at pressures up to 3000 psig to be analysed reliably without condensation of any hydrocarbons. The system basically encompasses a series of heated pressure regulators and a specifically designed heated sampling station. This retains the Amasil$^{TM}$ traps at an elevated temperature, such that mercury and organomercury compounds are retained on the trap and hydrocarbons purged to waste. Once a set volume of gas has been passed over the trap, the trap is removed and transferred to a commercially available atomic fluorescence instrument for measurement.

The pressure let-down system is transportable and can be easily coupled to existing sampling stations. Use of this device avoids problems associated with contamination and provides a steady stream of gas over the traps. Removal of a trap does not disturb the flow pattern and the next sample can be taken at will.

Experience with this system will be described in this paper, and results presented from a wide range of applications, both onshore and offshore.

### A novel approach for the determination of mercury in natural gas condensates using an atomic fluorescence spectrometry

*W. T. Corns, P. B. Stockwell, Azman Bin Shafawi[1], M. Foulkes[1] and L. Ebdon[1], PS Analytical, Arthur House, Crayfields Industrial Estate, Main Road, Orpington, Kent BR5 3HP, UK; [1]Department of Environmental Science, University of Plymouth, Drake Circus, Plymouth, Devon PL4 8AA, UK*

Mercury levels in the petrochemical industry are distributed in both the liquid and gaseous phases. It is therefore important to be able to quantify the levels of mercury in both liquid and gas streams reliably prior to the installation of removal procedures. Reliable systems for the measurement of mercury in natural gas are available, but few existed prior to this work for the analysis of mercury in condensates. Shafawi *et al.* [*Analyst* (1998), in press] have described a simple procedure for the measurement of the mercury in condensates. This paper will describe the configuration of a new accessory which links to the Sir Galahad atomic fluorescence instrument and allows the Hg levels in condensates to be measured.

The instrument comprises a vaporizing chamber and a sample collection system onto Amasil traps held at an elevated temperature. Samples of condensates are directly injected onto the vaporizing chamber and the vaporized mercury collected onto the trap. Argon- or mercury-free nitrogen are used to transport the vapour over the traps where the mercury or organomercury compounds amalgamate with the gold on the traps and the hydrocarbons are vented to waste. The heated traps efficiently retain the mercury whilst avoiding condensation of the hydrocarbons by virtue of being above the dew point.

Results will be presented in this paper which demonstrate the reliability of the accessory to analyse all forms of mercury in natural gas condensates. A range of condensates, covering a broad spectrum of mercury levels and sources of material, have been analysed and the results will be presented.

### Rapid, automated preparation of working standards and samples in flame atomic absorption spectroscopy

*David Pfeil and Malcolm Lee, Unicam Atomic Absorption, PO Box 207, York Street, Cambridge CB1 2SU, UK*

The technique of atomic absorption spectroscopy has a dynamic range of about two orders of magnitude. Because the dynamic range is so limited, analysts often must interrupt an analysis to perform additional sample dilutions. Such operator intervention negates the advantage of today's highly automated atomic absorption spectrometers.

In this paper, we will discuss design features of a new computer-controlled, in-line dilution accessory, designed to provide fast and accurate dilutions without operator intervention. Detailed data will be presented on the sequencing of such devices, the possible dynamic working range and repeatability of the technique and, of course, the achieved accuracy of such systems.

The accessory has been applied to the determination of real samples and typical results of in-line dilution will be compared with manually prepared dilutions.

### Simultaneous sulphur emission/selenium fluorescence detection in a hydrogen diffusion flame following flow-injection hydride generation

*Julian F. Tyson and Christopher D. Palmer, Department of Chemistry, Box 34510, University of Massachusetts, Amherst, MA 01003-4510, USA*

Modifications have been made to a Baird ICP-AFS instrument so that it now has the data acquisition speed to monitor transient chromatographic or flow-injection peak profiles. In addition to the ICP atomizer, a cold vapour cell (for mercury determinations), and an argon-hydrogen diffusion flame have been used as atom reservoirs. The instrument has been adapted to monitor simultaneously sulphur (by molecular emission) and selenium (by atomic fluorescence). The analyte elements, in a tetrahydroborate matrix, were merged with an HCl stream in a flow-injection manifold and, after gas–liquid separation, the analytes were introduced into an argon hydrogen diffusion flame in the form of hydrogen selenide and hydrogen sulphide. The hydrogen for the flame was generated from the reaction of acid with the excess tetrahydroborate. Currently, the limit of detection (3 s) for selenium is 10 μg/l, and for sulphide is 70 μg/l (200-μl sample, hollow cathode lamp source for selenium fluorescence). The calibration was linear for selenium to 2 mg/l, and to 100 mg/l for sulphur, when doublet peaks occur. The throughput is 180 injections per hour. Selenate can be reduced to the hydride-forming selenite, and sulphite to sulphide, by microwave-assisted, on-line reduction in the HCl carrier stream. The possibilities for the reduction of sulphate to sulphide in a flow injection manifold are being investigated, though only a limited number of reagents have been reported for this reduction. In addition to applications in the analysis of waters and wastes, a possible application of this system is as a detector for the HPLC separation of Se and S species extracted from selenium-enriched yeast samples, so that conditions which would avoid co-elution could be found. Mass spectral identification of minor Se compounds in the presence of high concentrations of co-eluting S compounds is likely to be difficult.

### Near-line process monitoring by laser-induced breakdown spectroscopy

*Gretchen E. Potts, Igor Gornushkin, Shane Claggett, Hossein Nasajpour, Benjamin W. Smith and James D. Winefordner, Department of Chemistry, University of Florida, Gainesville, FL 32611-7200, USA*

Phosphate rock mined in Florida has considerably decreased in quality in the past 10 years. Therefore, several fertilizer manufacturers are exploring new methods of process monitoring to improve product yield. To save money, poor quality rock has to be detected before it is sent to be chemically processed. At present, ICP-OES is the common analytical technique to monitor the rock. However, the analysis takes several hours. Laser-induced breakdown spectroscopy (LIBS) is being explored as a real-time alternative to this time lag problem.

In an effort to explore the possibility of utilizing LIBS for phosphate rock analysis, we studied several methods of data analysis. Though several forms of phosphate rock were studied, the best precision was obtained with pressed pellets. A computer program was written with Visual Basic to facilitate the data work-up.

Recently, a method for correction of variations in single-shot spectra obtained from soils and aerosols was proposed by Bulatov *et al.* [*Anal. Chem.*, **69** (1997), 2103–2108]. Each single-shot spectrum was considered and spectral line intensities were found to correlate with the continuum plasma background. Using this model for phosphate rock, a weak correlation was observed. In order better to understand and explain the results obtained, the model by Bulatov was explored using a sine function to simulate the fluctuations. Our models reveal that this method only accounts for plasma fluctuations and not concentration fluctuations which are present in heterogeneous samples, e.g. phosphate rock.

To describe both fluctuations better, Bulatov's equations were altered to account for concentration fluctuations which are independent of the plasma fluctuations. This single-shot spectra fits a normal distribution. Linear calibration curves were obtained with precisions of 5–10%. The accuracy of the rock samples was checked with a commercial ICP-OES.

In addition, a portable LIBS system has been assembled with an Ocean Optics Spectrometer and a Kigre Nd:YAG laser. Though it does not provide the spectral resolution of the benchtop system, it does offer the versatility of being portable. Several phosphate rock samples have been studied and similar precision has been observed.

### Determination of arsenic and selenium at sub ng/g level in semiconductor-grade sulphuric acid by hydride generation atomic absorption spectrometry with cold trap preconcentration

*Masashi Murakami, Masaru Ito, Shigeru Sasaki and Makoto Imai, Ehime Laboratory, Sumika Chemical Analysis Service, 3-1-39 Shinden-Cho, Niihama-Shi, Ehime 792-0003, Japan*

The demands on improving limits of quantification of metallic impurities in semiconductor-grade reagents have been expected. Therefore, we applied hydride generation atomic absorption spectrometry (HGAAS) with a cold trap preconcentration to the determination of arsenic and selenium, and achieved sub ng/g level determination in sulphuric acid.

The cold trap preconcentration system consisted of two U-shaped tubes made of borosilicate glass, and it was connected between the hydride generator (Perkin Elmer FIAS200) and the atomic absorption spectrometer (Perkin Elmer 3100) with silicone/Teflon tubing. An arbitrary volume of sample could be mixed into the carrier by the use of a T-connector. After the concentrating step, the second tube was moved to the heating net (arsenic) or hot water bath (selenium) to vaporize and introduce the trapped hydride to the furnace.

The sulphuric acid sample was diluted twofold for arsenic and fivefold for selenium measurement with ultra-pure water and hydrochloric acid. The introduced volumes were 10 ml for arsenic and 20 ml for selenium. The sensitivity was affected by the dilution factor of sulphuric acid, concentration of potassium iodide and hydrochloric acid. For selenium determination, it was also necessary to silanize the tubes and to stuff silanized quartz wool into the second tube. Limits of quantification for arsenic and selenium in the sulphuric acid were 0.02 ng/g and 0.1 ng/g, respectively. Relative standard deviations for 0.04 ng/g arsenic and 0.1 ng/g selenium were 11 and 7.1%, respectively. Arsenic and selenium content in a semiconductor-grade sulphuric acid were both below the limits of quantification.

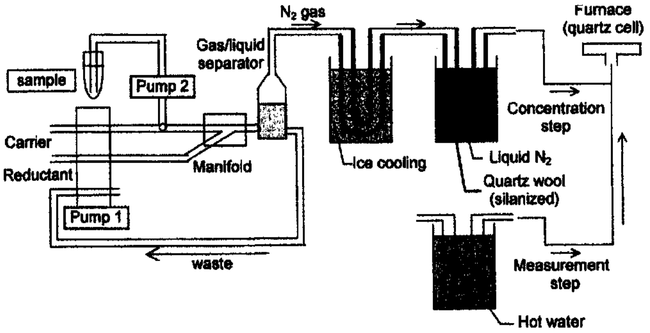

*Schematic of HGAAS with cold trap system for selenium.*

### Determination of As, Sb, Bi in geological samples with ICP-AES

*Qiu Li-Wen, Yu Lei[1] and Lu Bao-Yuan[2], State Key Lab. of Mineral Deposit Research, Nanjing University, Nanjing, 210093, P. R. China; [1]Department of Chemistry, Nanjing University, Nanjing, 210093, Nanjing, P. R. China; [2]Institute of Geology and Mineral Resources, Nanjing, 210016, P. R. China*

The three elements, As, Sb, Bi, as for Ag, Au, Cu, Hg, Mo, Pb, Sn, W, Zn, are important indicated elements in searching for the gold mine, which are usually determined by atomic fluorescent spectrometry (AFS). Because the conditions of their hydride generation are different and the contents among them have no proportion relation, they can not be determined simultaneously. And because the dynamic range of AFS is narrow, the samples must be diluted or weighed again if the content of a certain element is too high or low.

In this paper, the analytical method of determining As, Sb, Bi in geological samples by sequential ICP spectrometry is given. On the basis of work in AFS, the samples were decomposed with aqua regia (1:1), then determined using a JY 38S sequential spectrometer. The effects of major elements Si, Fe, Al, Ca, Mg, etc. on the spectral lines of As, Sb, Bi were studied. Because the most sensitive spectral lines of As, Sb, Bi are close to 200 nm, the effect of background Al is serious. Two ways to clear up the effect are studied: background subtraction; and interference coefficient correction. Simultaneously, the optimum conditions of the instrument are fixed. By using this method, the results of As, Sb, Bi (containing Cu, Pb, Zn) satisfy the quality requirements of geological research and searching for mineral deposits by chemical prospecting.

### Measurements of mercury concentration in cigarette and tobacco smoke

*Yury G. Tatsy, Yury I. Stakheev, Igor V. Bezsudnov and Vladimir A. Sevryukov, Vernadsky Institute of Geochemistry and Analytical Chemistry, 19 Kosygin Street, 117334 Moscow, Russia*

Mercury is concerned with number rationed elements—ecotoxicants, and is a subject of obligatory control in the environment. Mercury finds its way into the human body with food, drinks, toothpaste, cosmetics and inhaled air. The FAO and WHO recommendations state that mercury supply with food should not exceed 0.3 mg per week, and the Russian minimal risk level (MLR) for mercury in the air of a populated area is 0.3 $\mu g/m^3$.

The presence of mercury in cigarette and pipe tobacco, as well as in cigarette paper is a source of additional mercury supply in the smoker's body. For quantitative determination of mercury inhaled by smoking, several types of cigarettes were analysed. The procedure for measurements of mercury in smoke, paper, tobacco and ash was developed. For measurements in smoke, this procedure was maximally approximated to real smoking. Detection of mercury was performed by a portable atomic fluorescence mercury analyser with preconcentration on bispiral gold collector. The detection limit was 1 pg Hg, RSD < 0.1.

The obtained results show that the concentration of mercury in inhaled air by smoking essentially exceeds the MLR in air of a populated area. At the beginning of cigarette burning, the filter absorbs up to 20% of mercury vapours, and then its efficiency decreases up to 4–6%.

### Distributing customer data using secured websites

*Farai L. Rukunda, Daniel J. Wagel, Joseph G. Solch, Thomas O. Tiernan and Roger K. Gilpin, Brehm Laboratory, Wright State University, Dayton, OH 45435, USA*

Many laboratories have adopted e-mail for delivery of analytical results, and some have developed websites in an effort to advertise capabilities. Few laboratories, however, have realized the advantages secure private website areas can provide for managing customer data.

The recent advances in the quantity and quality of tools available for designing and operating World Wide Web (WWW) sites and the availability of low-cost web-hosting services have now made it both practical and advantageous for laboratories to manage their own sites. Modern operating systems and website software can easily handle the requirements for secure password-protected or encrypted access. This permits successful implementation of the secure submission and retrieval of customer data from private website areas that is essential to acceptance of this method of communication.

Easy to use software tools are available to design and manage websites maintained by commercial web-hosting services. Laboratories can also capitalize on the use of existing business tools by building websites that are designed to use standard word processors and spreadsheets to add and update data for customers. This approach permits the laboratory to control creation of the content of the website in-house but leaves the 24 h × 7 day website management and operation to a firm equipped to provide backups, a fast Internet connection and other necessary features.

This work presents the design, implementation and administration of two controlled access websites that were set up to provide both marketing information and up-to-date secure access to analytical data for multiple customers. Design considerations including a discussion of whether to host your own website will be presented in this paper. Good choices for operating systems and website software, hardware and software options, and typical set up and operating costs will be detailed. In addition, we will illustrate a number of implementation and operation problems that must be considered to provide a secure and efficiently run website.

### Automated monitoring of environmental procedures

*Robert Pavlis, Labtronics, 95 Crimea Street, Guelph, Ontario, Canada N1H 2Y5*

Many analytical techniques are affected by environmental conditions, e.g. temperature and humidity. As concerns for meeting laboratory guidelines and ISO 9000 standards increase, so does the need to monitor and record these elements.

The term 'environmental parameter', as used here, includes any parameter that can be measured automatically. This could be natural physical parameters, e.g. temperature, humidity and barometric pressure, or it may refer to chemical agents. The environment location could be a complete lab, a storage device, e.g. a refrigerator, or even a specific piece of equipment, e.g. a water bath.

The typical solution for an automated monitoring system consists of having a computer near each monitoring device. This is not cost effective and requires a large amount of computer support.

A new network-based system is presented that is able to use the standard computer network to monitor a complete facility from one computer. This new technology allows you to connect RS232 instruments directly to the network. Data are transferred through the network to any selected computer(s). Special software monitors the stations and saves environmental data to specific databases, as required by the customer.

Data can be reviewed and reported as required. Environmental data can be combined with instrument results before downloading to a LIMS. This allows the user to add temperature and humidity values to chromatography results, e.g. or to correct hardness values for humidity. The software can also monitor values and take action when they are outside of specified limits.

The resulting system requires virtually no maintenance, except for the actual monitoring devices. The software is password protected and can be easily secured.

### GC on-line probe for GC-MS process monitoring applications

*Wolf Muenchmeyer and Andreas Walte, WMA Airsense Analysentechnik GmbH, Hagenowerstr. 73, D-1 9061 Schwerin, Germany*

For process monitoring applications with a mass spectrometer (MS), fast results are essential. A direct connection of the MS with the process is also often required. The flexible on-line probe, which is basically a special gas chromatograph, is constructed for the direct introduction of gases from the process into a mass spectrometer. It is based upon a steel capillary for chromatography and is heated resistively at a programmable rate. The probe is processor controlled. It can be connected to any mass spectrometer by using a hot membrane inlet system.

With the heated probe head it is possible to sample high-boiling compounds, e.g. PAH. No condensation of water occurs when hot stack gases are sampled. A stainless mesh at the top prevents pollution by particles. Additionally, with the hot probe head, thermal desorption of surfaces, e.g. for the analysis of pesticides on wood, with a subsequent temperature programmed GC run can be carried out.

Different applications using a quadruple mass spectrometer (HP MSD) are shown in this paper.

### Automated SPE of tricyclic antidepressants

*Michael Urban-Piette, Lynn Stroede and Gary Lensmeyer[1], Gilson, Middleton, WI 53562, USA; [1]Clinical Toxicology Laboratory, University of Wisconsin Hospital, Madison, WI 53792, USA*

Antidepressant drugs have represented $8.7 billion in sales for 1997. Of these drugs, the tricyclic antidepressants (TCAS) are the oldest class with imipramine (tofranil) having been introduced in the early 1950s. Physicians monitor patients' serum TCA concentration to minimize cardiac arrhythmia and hypotension, common side effects of this class of drugs. Rapid and efficient test assays are therefore required. Here we report an automated solid-phase extraction (SPE) method for monitoring the TCAs in serum. Unique to this process is the use of thin SPE disks and a new automated instrument to process the samples prior to HPLC analysis.

Method performance studies demonstrated a between-run coefficient of variation (CV) less than 15%. The lowest limit of detection was 10 ng/ml from 1 ml serum. Recoveries ranged from 70 to 98% and the assay was linear to at least 1000 ng/ml.

Several parameters were studied during optimization of the method. Specifically, recovery was found to be inversely related to the acetonitrile content in the wash solution. Also, recovery approached a maximum at a threshold volume of butylamine in the eluting solution.

The high sensitivity of the method can be attributed to the extraction disk technology which afforded decreased elution volumes. Most important, the new automated process allowed optimized sample throughput of at least 51 samples/h.

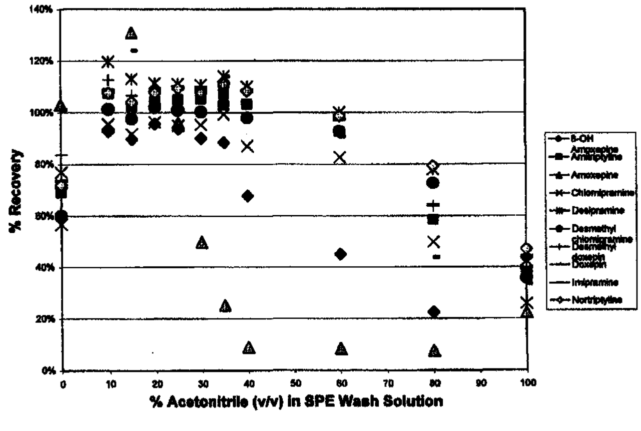

*Recovery as a function of % Acetonitrile (v/v) in SPE wash solution.*

### Gas diffusion-flow injection system with preconcentration for the analysis of fluoride in serum and semen

*Lucía Hernández-Garciadiego, Humberto Gómez-Ruiz, Pilar Cañizares Macías, Fernando Díaz-Barriga*[1] *and María Deogracias Ortiz-Pérez*[1]*, Facultad de Química UNAM, Ciudad Universitaria, México, D.F. 04510, México;* [1]*Facultad de Medicina UASLP, Av. Venustiano Carranza 2405, San Luis Potosí 78210, S.L.P., México*

Fluorine is one of the elements whose determination is subjected most to interference from other species (formation of metal/fluoride complexes or insoluble salts) For this reason, separation of fluoride prior to its determination is commonplace. Separation of fluoride by distillation is time consuming, so new methods are aimed at overcoming the need for it.

Serum and semen samples contain low levels of fluorine, and it is necessary that the sample's volume does not exceed 3 ml. So, the two main goals in this study are the determination of low levels of fluorine in low-volume samples.

In this paper, a method for the fluoride determination based on the conversion of fluoride to the volatile trimethylfluorosilane (TMFS) using hexamethyldisilazane (HMDSA) in acidic medium is reported. TMFS evaporates and diffuses through a polypropylene membrane to be absorbed into a sodium hydroxide acceptor stream at the upper part of the diffusion cell. A fluoride-selective electrode then determines fluoride. For the fluoride determination at low levels, an open-closed configuration is proposed. The volatile TMFS leads to the lower part of the gas diffusion cell and diffuses through the membrane to be absorbed in the stationary acceptor stream enclosed in the sample loop of a second injection valve, where it is preconcentrated.

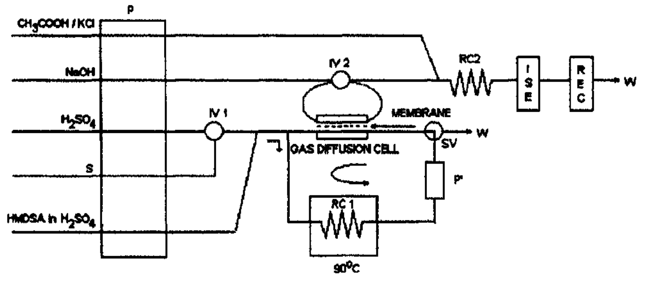

*Manifold for the fluoride determination. Preconcentration model.*

### Automatic calibration for the non-enzymatic spectrophotometric determination of reducing sugars without pre-treatment of the sample

*Ma. del Pilar Cañizares-Macías, Lucia Hernández-Garciadiego and Humberto Gómez-Ruíz, Facultad de Química, UNAM, Ciudad Universitaria, México, D.F. 04510, México*

A non-enzymatic spectrophotometric method coupled to an automatic system of standard additions, to determine the reducing sugars, based on the reaction with 3,5-dinitrosalicylic acid (DNS) is proposed. In a system based on an exhaustive reaction, sample volumes are introduced together with calibrations solutions, and thus the calibration is performed in a non-segmented flow system. Homogenization of the sample/standard/carrier takes place in the calibration loop. Later it is injected in the flow injection analysis (FIA), where the reaction with the 3,5-dinitrosalicylic acid, to obtain 3,5-diaminosalicylic acid measured spectrophotometrically at 480 nm, is carried out. This method allows a direct analysis of liquid samples with no pretreatment and avoids matrix effects. With the proposed FIA method, a greater sample throughput is obtained, 11 samples/h, and accuracy, expressed as relative standard deviation (RSD), is 2.3%.

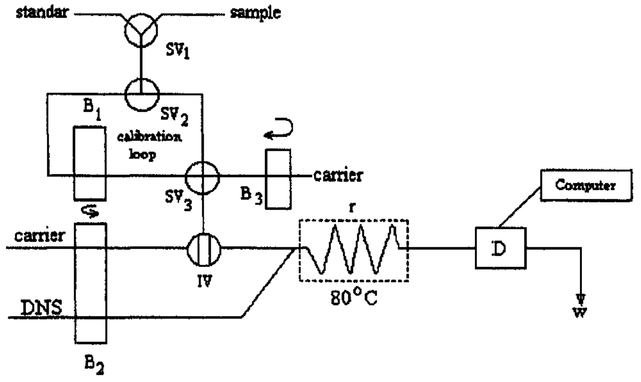

*Manifold for the determination of reducing sugars.*

### On-line analysis of peroxides in disinfectants

*Diedrich Harms, Roberto Than, Bernt Krebs and Uwe Karst, Anorganisch-Chemisches Institut, Abteilung Analytische Chemie, Westfälische Wilhelms-Universität Münster, Wilhelm-Klemm-Str. 8, D-48149 Münster, Germany*

In recent years, peroxides have gained popularity as disinfectants and bleaching agents in industrial and household application due to their environmentally beneficial properties. As oxidation is the active process, hydrogen peroxide and peroxyacetic acid (PM) yield water and acetic acid, respectively, as residues of the disinfection. However, to minimize costs and to ensure consumer protection, the content of the peroxides in solution has to be determined frequently.

We will present highly selective methods for the determination of hydrogen peroxide and PAA. Hydrogen peroxide reacts with a dinuclear iron(III) complex forming a coloured adduct which can be detected by means of UV/vis spectroscopy in the range between 560 and 600 nm. A flow-injection analysis system has been set up for on-line analysis of hydrogen peroxide in the concentration range from 5 to 500 µmol/l. The interference by other peroxides is negligible. Up to 60 samples can be analysed per hour. Additionally, a flow-injection system for the determination of PAA in the concentration range between 2 and 500 µmol/l has been constructed. ABTS, a common peroxidase substrate, is oxidized by PM in the presence of iodide as catalyst, but without peroxidase, to a green-coloured radical cation with absorption maxima between 405 and 815 nm. Both methods have been validated by independent methods, including liquid chromatographic techniques.

### Automated SPE method development using a dedicated system for hyphenated SPE

*O. Halmingh and J. A. Ooms, Spark Holland B.V., P.O. Box 388, 7800 AJ, Emmen, The Netherlands*

As a result of the introduction of combinatorial synthesis and high-throughput screening in drug development, the number of drug candidates reaching phase I, II and III clinical studies is increasing rapidly. As a consequence, the bioanalytical labs in the pharmaceutical industry are focussing on increased speed of sample clean-up and analysis. Obviously, high-throughput analysis will shorten the time between different assays, and therefore rapid method development for SPE clean-up and analysis of biological samples is becoming crucial to maintain efficiency of high-throughput analysis.

The development of a solid-phase extraction (SPE) procedure involves the optimization of a considerable number of variables, e.g. sorbent type, solvent type and volume for extraction, for clean-up and desorption, sample extraction capacity, flow rates, etc. Traditionally, these variables are optimized by measuring the recovery of the analyte as a function of the variable. A major problem with this approach is that it gives little insight into the various possible causes for incomplete recovery, e.g. breakthrough, adsorption to tubing or glassware, and degradation (light, oxidation, etc.). Therefore, SPE method development is often a tedious trial-and-error procedure rather than systematic optimization. Plus, it remains uncertain whether the final method is the optimum one.

We have developed a strategy for rapid SPE method development, which monitors the entire extraction process with respect to analyte loss. For every single extraction experiment, recovery is automatically measured together with analyte loss caused by adsorption, degradation and breakthrough. The strategy is based on the use of a PROSPEKT system with a dedicated configuration for method development based on serial SPE cartridge processing. In addition, a new sorbent has been developed and evaluated for use as generic sorbent for fast method development of SPE-LC/MS analysis of biological samples.

### Performance of a next generation vial autosampler for the analysis of VOC in water matrices

*Ed Price, Tekmar-Dohrmann, 7143 E. Kemper Rd., Cincinnati, OH 45249, USA*

In today's laboratories, increased efficiency and productivity is of extreme importance. Equally important is the ability to automate analyses without sacrificing sample integrity or data quality. A new vial autosampler, the AQUAtek 70, has been developed to fully automate purge-and-trap analysis of water samples in accordance with current EPA methods for volatile analysis.

The AQUAtek 70 liquid autosampler is a 70-position autosampler that can handle water and wastewater samples of all types including particulate laden samples. The AQUAtek 70 offers improved data quality with automatic sample volume measurement and automatic standard addition. A high-temperature OptiRinse system virtually eliminates carryover and improves productivity.

Research will be presented demonstrating the instrument's ability to transfer sample aliquots with the addition of internal standard or surrogate solutions. Data will be evaluated for linearity, precision and accuracy. In addition, the instrument's sample pathway will be evaluated for carryover, innertnesss and reliability.

### An automated system for focused microwave-assisted digestion of samples

*Jannell M. Rowe, Robert J. Fix Sr and Charles B. Rhoades Jr, R. J. Reynolds Tobacco Company, Bowman Gray Technical Center, P.O. Box 1487 Winston-Salem, NC 27102-1487, USA*

Sample preparation is the most time-consuming and labour-intensive step in the process of elemental analysis. Microwave digestion using concentrated acids to prepare samples for analysis has done much to alleviate this problem. Closed-vessel microwave systems capable of operating at elevated pressures and temperatures reduce the time required for sample decomposition. However, sample size is typically limited to 500 mg and reagent addition during the digestion process is difficult. Open-vessel microwave digestion systems allow for a larger sample size and reagent addition during digestion.

The STAR System-focused microwave from CEM Corporation is an open-vessel digestion system with two or six microwave cells. Each cell has its own temperature sensor allowing individual temperature feedback control. Automated reagent addition, vapour containment, and operation at atmospheric pressure enhances the safety and flexibility of this system.

A PC-based control system has been integrated with the STAR System 6 allowing unattended preparation of up to 59 samples. The system's Visual Basic program allows the analyst to automatically load and unload samples utilizing an XYZ gantry equipped with a gripper. The software also allows the analyst to control digestion parameters, e.g. number of samples processed, time and temperature of reaction, reagent addition, and final dilution. Automating the STAR System 8 with an integrated control system allows for rapid, less labour-intensive sample preparation and increases sample throughput while improving reproducibility.

### Utilization of an automated coagulation analyser for determination of coagulation factor activities in biological product formulations

*Juan Torres, Marsha Richmond, Tonya Barnes, Kathy Meyer and Annjanette Best, Bayer Corporation, Pharmaceutical Division, Biological Products, P.O. Box 507, 8368 US 70 West, Clayton, NC 27520, USA*

Advancements in clinical diagnostic instrumentation are allowing for increased placement of fully automated testing systems into the QA/QC laboratory. These automated instruments minimize sample preparation time and increase assay throughput. For coagulation products manufactured from human plasma, determination of individual coagulation factor activities is critical in assessing the final product efficacy.

An Electra 1600C automated coagulation analyser was evaluated for the determination of factor II, VII, VIII, IX and X in production intermediates and formulated final product. Factors II, VII and X were measured using the prothrombin time, which measures the response of the extrinsic coagulation system. Factors VIII and IX were measured using the activated partial thromboplastin time, which measures the response of the intrinsic coagulation system. Activity levels are interpolated by comparing the clotting times of various dilutions of test material with a standard curve.

Comparison studies were conducted between a coagulation analyser requiring manual sample preparation and reagent addition and the 'automated' Electra 1600C. Introduction of such an automated system into the QA/QC environment provides for execution of multiple testing protocols from one sample without requiring manual sample preparation. The fully automated system provides increased precision without loss of accuracy.

### Evaluation of an automated clinical chemistry analyser for implementation into a biological products QA/QC laboratory

*Juan Torres, Marsha Richmond, Jeff Bumgarner and Franz Gruswitz, Bayer Corporation, Pharmaceutical Division, Biological Products, P.O. Box 507, 8388 US 70 West, Clayton, NC 27520, USA*

Often established testing methods utilized by QA/QC pharmaceutical laboratories can be labour intensive and time consuming. Many of these methods require extensive sample preparation and result in insufficient testing periods. In order to limit the amount of time required for manual sample preparation, an automated clinical chemistry analyser has been evaluated for use in our laboratory. The 'opeRA®' chemical analyser manufactured by Bayer Diagnostics Division is being evaluated for testing of biological products. Parallel testing was performed with current standard methods and the automated opeRA® system.

Comparison studies between the current test methods and the automated opeRA® system to investigate precision, accuracy, linearity and detection limit were conducted using product and control samples. All product and control samples used in these studies were derived from human plasma. A projection has been made of the benefits provided by the automation of these assays using the opeRA®.

### Automated fluoride analysis

*Mark Derikx, Labtronics, 95 Crimea Street, Guelph, Ontario, Canada N1H 2Y5*

A new automated fluoride analysis system makes use of modern flow-through ISE technology. This new electrode design results in shorter stabilization times and less carryover.

The electrode allows samples to flow through ion-selective material, essentially eliminating dead volume. Surface area contact between the sample and the ion-selective material is maximized. Data will be presented in this paper to compare the stabilization times of this new technology to conventional electrodes.

A complete system that combines this electrode with an automated flowing system is presented. The automated fluoride system transfers samples, adds adjusting buffer and carries out the complete analysis procedure. Blank corrections and carryover corrections allow the system to measure very low levels of fluoride.

Electrode technology can be extended for use with other types of solid-state ISE electrodes.

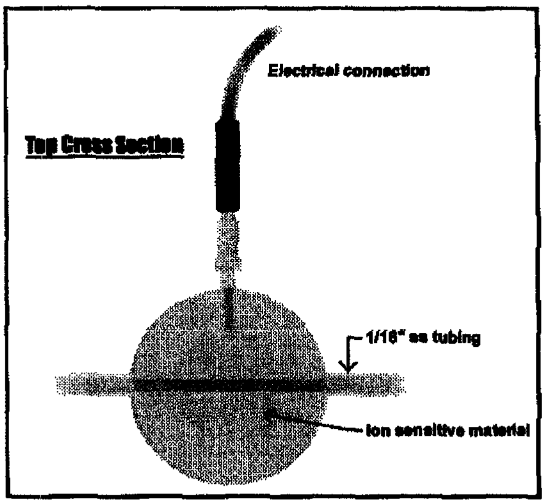

### Automated calorimetry system

*Robert Pavlis, Labtronics, 95 Crimea Street, Guelph, Ontario, Canada N1H 2Y5*

Hundreds of different manual tests have been developed that produce a coloured end point, which can be measured with a calorimeter, spectrophotometer or fluorometer. For a number of analyses, it is important to measure other parameters along with the absorbance measurement. For example, pH can affect the colour of samples, making it difficult to accurately evaluate concentration levels.

A new automated system will be presented that can measure from three different instruments simultaneously. This system is able to measure the absorbance and pH of a sample, e.g. and then correct the absorbance for pH.

Other examples will be presented that combine three unrelated instruments into a single analysis system. Beer can be analysed with a calorimeter, density meter and a pH meter simultaneously. Sugar samples can be analysed with both a polarimeter and refractometer at the same time.

The software component of this new automated system can be configured to collect data from any analogue or

RS232 detector. It is able to control robotic samplers, pumps, water baths, dilutors and most simple instruments. A very simple user interface allows one to configure the steps in an experiment. The complete system can perform sample preparation, dilution, reagent addition and sample analysis.

The most unique feature of the system is its ability to incorporate existing detectors. Rather than buying a 'beer analyser', the software allows you to create a beer analyser using the best analytical components available.

### Analysis of five HIV compounds in human plasma using automated SPE and LC/MS

*John P. Walsh, John A. Begley, John P. Macneela and Bernhard M. Lampert, Triangle Pharmaceuticals, 4 University Place, 4611 University Drive, Durham, NC 27707, USA*

Four nucleoside reverse transcriptase inhibitors (RTIs) and a protease inhibitor were co-administered to human patients for a drug interaction study. The protease inhibitor, DMP-450, is a cyclic urea that possesses unique physical properties compared to AZT, 3TC, ddl and d4T. This made a single short isocratic LC/MS procedure impractical. Therefore, a two-stage automated solid-phase extraction and two short (3.2 min run time) LC/MS/MS procedures were developed.

Automated sample preparation was accomplished on a Zymark RapidTrace SPE Workstation. Elution of the RTIs in 20% acetonitrile preceded an elution of DMP-450 with pure acetonitrile. Each stage of the elution had an internal standard that traced the respective analytes through analysis.

In the first short LC/MS/MS procedure, separation of the RTIs was attained with a Keystone Scientific Kromasil C18 HPLC column with an acetonitrile/0.05% TFA in water mobile phase. Quantitation of the compounds was performed on a Micromass Quattro LC in the MRM mode. In the second LC/MS/MS procedure, DMP-450 was separated from

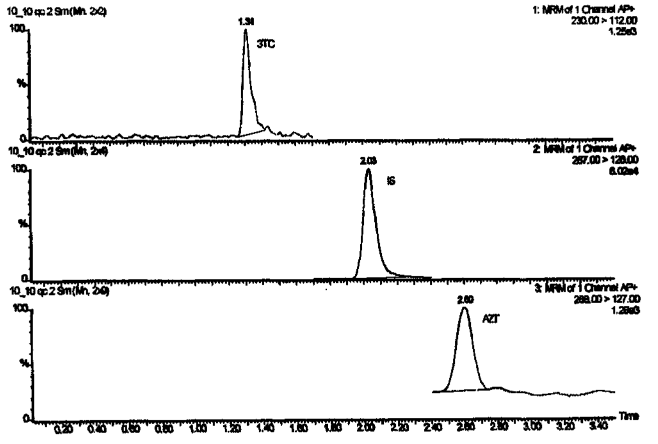

*Typical reconstructed ion chromatogram of a human plasma sample fortified with 10 ng/ml of 3TC and AZT.*

endogenous plasma compounds on a Keystone Scientific Betasil phenyl column and detected with the Finnigan LCQ ion trap in the MRM mode. The implementation of two different methods on separate instruments allowed the samples to be analysed in a timely fashion. Both methods proved to be fast and rugged and were used to quantitate all compounds with a LOQ of 10 ng/ml.

### Rapid identification of metabolites using an automated LC-MS/MS system

*Joseph F. Anacleto, John Robson, Eva Duchoslav and Ron Bonner, Perkin-Elmer Sciex Instruments, 71 Four Valley Drive, Concord, Ontario, Canada L4K 4V8*

Identification of drug candidates requires the rapid evaluation of biological activity, biological availability, pharmacokinetics and characterization of metabolites. The ever-increasing number of potential lead compounds generated by combinatorial chemistry and high-throughput screening requires efficient methods for selecting and further developing suitable candidates.

Metabolism samples, whether from animals or model systems, are generally very complex and contain high levels of endogenous material. The favoured approach to characterization of metabolizes is to use a triple quadruple LC/MS/MS system to obtain good quality product ion spectra which can then be used to identify potential metabolites. The analytical procedure traditionally employed for the determination of metabolites typically involves the preliminary analysis of the sample by full scan MS followed by interpretation of the data to identify potential metabolites. One method used for identification of the metabolites is to simply look for peaks in the extracted ion chromatograms (XIC) of predicted common metabolites. For example, many compounds form oxygenated metabolites; by examining the XIC corresponding to the mass of the parent drug plus 16, it is possible to quickly locate any oxygenated metabolites. Once the potential metabolites have been located in the chromatogram, an LC/MS/MS product ion method is built, the MS/MS data acquired and then interpreted to determine the structure of the metabolites. This approach, whilst very successful, can be time consuming both in locating potential metabolites and in building the LC/MS/MS methods.

In this paper, we describe the development and application of new software and hardware for the automated analysis of metabolites in biological samples. The LC/MS/MS system can be used for automatically locating expected metabolites in an LC/MS analysis. The system will then generate a method capable of recording the product ion spectrum of each potential metabolite identified. While primarily intended for use in drug discovery, the system can also be used to automate the more rigorous analysis required during drug development.

### Online measurement of oil compounds in the exhaust gas of combustion engines by mass spectrometry

*G. Matz, W. Schröder, M. Gohl and A. Walte*[1]*, Technical University of Hamburg-Harburg, Harburger Schloss-Str. 20, D-21079 Hamburg, Germany;* [1]*WMA Airsense Analysentechnik GmbH, Hagenower Str. 73, D-19061 Schwerin, Germany*

To reduce the emission from combustion engines, the control of lubricant evaporation is necessary. For a better understanding of the process causing oil loss by emission, every phase of the combustion cycle as well as the engine operation during different driving cycles has to be monitored on-line.

The on-line measuring system under development is based on the RHQ-MS (round hyperbolic quadruple mass spectrometer) (Bear Instruments) equipped with a high-temperature direct inlet. The MS consists of an EI/CI source, a collision cell and four hyperbolic quadruples in a cyclic arrangement. The inlet conducts the 800 °C hot exhaust gas directly from the combustion chamber via a skimmer to the ion source of the MS. Operating in SIM mode with chemical ionization it is possible to monitor the concentration of various compounds of the exhaust gas in real time (1 ms).

Analyses on an engine test stand with a methane-powered motor operating with different lubricants have been carried out. On-line measurements of exhaust gas components ranging from gaseous to low volatile compounds emitted during realistic engine working cycles have proven that dynamic processes can be monitored with a time resolution of a few ms.

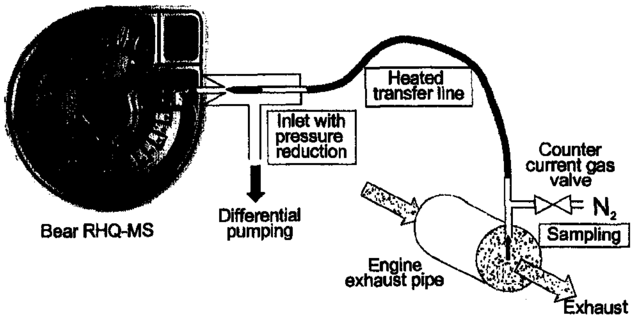

*Fast online analysis of engine exhaust compounds.*

### Automated electrochemistry with mercury drop electrode

*H. G. Jayaratna and C. S. Bruntlett, Bioanalytical Systems, 2701 Kent Avenue, West Lafayette, IN 47906, USA*

A simple design of a flow-cell for the conventional mercury drop electrode (MDE) and its use with an autosampler are described.

Trace metal analysis by anodic stripping voltammetry (ASV), the popular application of MDE, or other techniques can easily be performed in an automated fashion. The present cell design is made of polycarbonate material and allows the use of standard Ag/AgCl reference and stainless steel auxiliary electrodes.

Complete automation was achieved with a 30-vial autosampler. A peristaltic pump was used to deliver samples to the cell. A 1.5-ml portion of the sample or the blank was placed in each vial. The volume of the cell cavity was ~70 μl. The cell was tested for its ability to reproduce electrochemical data. Sample throughput depends on the technique, the concentration of the analyte (deposition time) in the case of stripping voltammetry and the efficiency of cell flushing. Data from stripping voltammetry of trace metals will be presented in this paper.

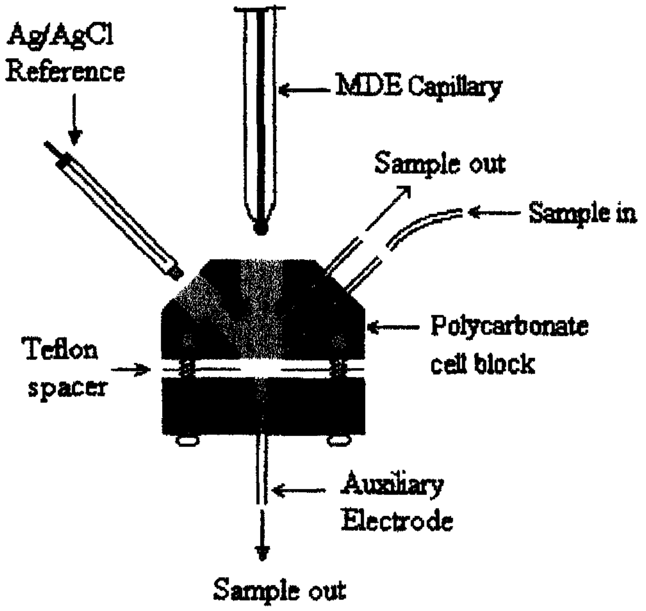

*Schematic of the MDE flow-cell arrangement. MDE capillary and the reference electrode were fitted with conventional plastic LC fittings.*

### Pattern recognition of organophosphorus pesticide and chemical agent infrared spectra

*H. T. Mayfield, Delyle Eastwood*[1] *and L. W. Burggraf*[1]*, Air Force Research Laboratory, Airbase and Environmental Technology Division, 139 Barnes Drive, Suite 2, Tyndall AFB, FL 32403-5323, USA;* [1]*Air Force Institute of Technology, Department of Engineering Physics, 2950 P. Street, Wright Patterson AFB, OH 45433, USA*

A collection of infrared spectra of various organophosphorus pesticides, military nerve agents and other organophosphorus compounds was assembled and analysed by pattern recognition and other chemometric techniques. The spectra were obtained as machine-readable files from a variety of sources including the US Army CBDCOM, military contractors and commercial spectral libraries. The spectra were imported from their various native file formats to a common file format. The resulting spectra were subjected to various preprocessing techniques in order to remove artefacts arising from the variety of collection sites and conditions, and were then transduced to produce data vectors representing the spectral range from 650 to 2500 reciprocal cm.

An important distinction to be made with the data was the discrimination between civilian pesticides and military nerve agents. Also, because the new chemical warfare convention requires monitoring for precursors and hydrolysis products of chemical warfare agents, the military nerve agents were combined in a single class along with their precursors and hydrolysis products. Autoscaling and Fisher weighting techniques were applied to the spectra to optimize the classification. Various classical pattern recognition techniques as well as neural network classifiers were investigated and compared in terms of classification accuracy. Various wavelength 'bin' sizes were investigated in order to establish the minimum spectral resolution necessary for satisfactory classification of the spectra. The effects of the removal of water-absorbing wavelengths and other potential spectral interferences were also investigated.

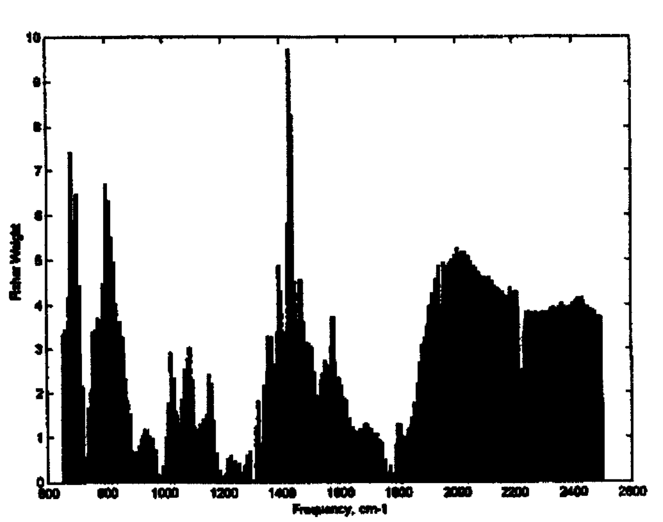

*Plot of Fisher weights versus frequency for the pesticides versus nerve agents. Data were transduced into 200 spectral bins.*

### The use of software wizards and on-line tutorials to educate users and enhance productivity in infrared spectroscopy

*Mike Fuller and Todd Clermont, Nicolet Instrument Corporation, 5225 Verona Rd., Madison, WI 53711, USA*

Today's analytical laboratory is filled with a myriad of computerized analytical instrumentation. Generally, each device is controlled and the data analysed by proprietary software developed by the equipment manufacturer. Having to learn how to operate all of these different software programs is quite a burden for the bench chemist. Over the last 2 years we have been involved in the development of function wizards and multimedia tutorials to be used with a commercial FT-IR spectrometer. The wizards guide the user in the creation of spectral libraries and provide suggestions for paths of development in quantitative analysis. Using these wizards allows relatively novice users to complete complex tasks. Multimedia tutorials can provide an on-line tutor to explain how the software and hardware operate, and provide the information needed to choose the correct sampling approach and accessory.

### Monitoring TOC in various drinking water sources

*Jay D. Rasmussen, Michael E. Potts, Karen Clark and John R. Stillian, Anatel Corporation, 2200 Central Avenue, Boulder, CO 80301, USA*

Municipal water systems are confronted with a variety of problems in treating contaminates in the water supply, Congress enacted in 1974 the Safe Drinking Water Act for the purpose of protecting the drinking water supplies. This Act, which is regulated by the Environmental Protection Agency (EPA), sets maximum limits for microbiological, radiological, physical and chemical contaminates. Monitoring the amount of total organic carbon (TOC) in drinking water is a good test for determining water quality. Analysis of water for specific compounds is time consuming and expensive. Total organic carbon analysis can be employed to help monitor water treatment practices by measuring for organic by-products of the disinfection process.

A reliable, cost-effective, TOC analyser is an invaluable tool in controlling water quality. A new TOC analyser is now available which uses a sequential injection analysis (SIA) design combining a multi-port switching valve with a syringe pump to accurately mix reagents and sample. The organic compounds are oxidized to carbon dioxide by UV/persulphate oxidation and the non-dispersive infrared (NDIR) detector measures the carbon dioxide concentration. This instrument is designed for both on-line and laboratory applications.

Due to consumers concerns regarding the quality of water from municipal water systems, the sales of bottled water continue to increase. The Food and Drug Administration (FDA) classifies bottled water as a 'food' and requires every bottled water product to be analysed under the provisions of the Safe Drinking Water Act. The FDA has established standard definitions used to identify the source of the bottled water. These definitions 'artesian', 'well', 'mineral', 'spring', 'purified', 'distilled' and 'sparkling' are used in labelling the bottled water. Some states also require additional testing as a condition to sell bottled water in the state.

Several kinds of bottled water representing domestic and international sources were analysed to determine the TOC concentration. Results are presented to compare the TOC levels in a variety of sources of bottled water from various regional areas.

### *In situ* analysis of residues formed on the chamber surface using infrared reflection absorption spectroscopy and electrostatic quadruple plasma analyser

*Hiroki Kawada, Hiroyuki Kitsunai and Nobuo Tsumaki, Mechanical Engineering Research Laboratory, Hitachi, Kandatsu 502, Tsuchiura, Ibaraki, 300-0013 Japan*

Residues on the inner surface of an electron cyclotron resonance (ECR) dry etching process chamber using microwave and magnetic coils, were *in situ* investigated with infrared reflection absorption (IRA) spectroscopy and energy selective mass spectrometry. The residues, which cause particle contamination during the dry etching process, were identified with the IRA spectra obtained by a specially designed optical set up. The chemical reaction of these residues was presumed by energy-analysis of incident molecules and ions obtained by electrostatic quadruple plasma analyser (EQPA) of Hiden Analytical Limited. Only a few minutes of the etching process is required for the above analysis. The infrared light of IRA spectroscopy was reflected on residues sticking on $SiO_2$ film formed on a mirror sample which is mounted on the chamber surface. The incident molecules and ions were extracted through a pin hole in the process chamber in order to prevent plasma penetrating into the EQPA instrument.

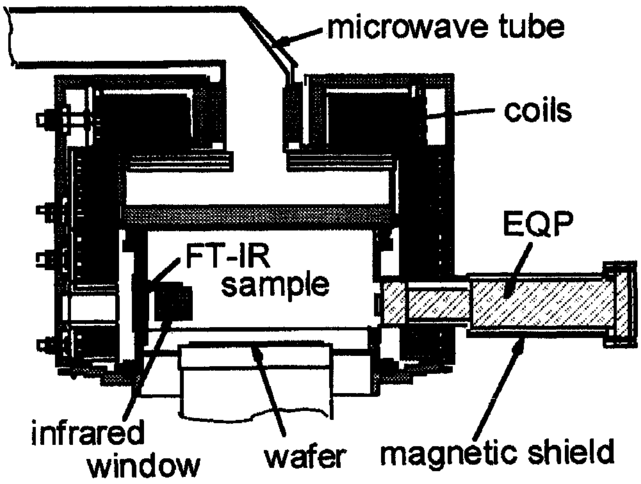

*Schematic of the analysis chamber equipped with EQPA.*

### Measurement of environmental polluting substances and in-line calibration of sensors using a special sampling probe

*Dieter Beckmann, Elmar Weckenbrock, Eugen Most, Heinrich Stöber, Uwe Spohn*[1] *and Bodo Fuhrmann*[1]*, Institut für Bioprozeß- und Analysenmeßtechnik e.V., Rosenhof, D-37308 Heiligenstadt, Germany;* [1]*Institut für Biotechnologie, Martin-Luther-Universität Halle, Kurt-Mothes-Str. 3, D-06120 Halle, Germany*

Because of its economic procedure, the flow injection analysis is more and more preferred by many laboratories. To make the advantages of flow systems usable for the field analysis, the development and testing of a double capillary probe at the base of flow injection analysis is presented.

It consists of two centred capillaries of different diameters. By adjustment of different flow volumes inside every capillary, it can be realized that either a sample is taken from the analyte and injected into a carrier supplied through the outer capillary or a calibration standard can be supplied to a sensor inside or behind the inner capillary. Therefore, it is possible either to measure the concentration of an analyte with or without a sample conditioning or to adjust the sensor in-line.

This paper shows that the detection of peroxides on the base of chemiluminescent reactions leads to detection limits in the lower micromolar range with linear calibration curves of four decades.

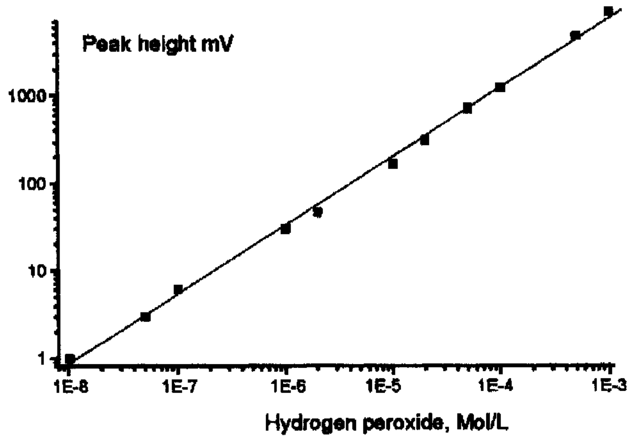

*Calibration curve for the chemiluminescent detection of $H_2O_2$.*

### On-line process monitoring model for electroless gold plating bath components

*Anita Sargent and Omowunmi A. Sadik, Department of Chemistry, State University of New York at Binghamton, Binghamton, NY 13902-6016, USA*

Electroless gold plating is used in the production of printed circuit boards due to low contact resistance, ability to plate evenly across minutely detailed junctions, and minimization of instrumental costs and maintenance. The electroless gold bath utilizing dimethylamineborane (DMAB) as reducing agent has become very attractive for this application because the reduction of gold occurs on a variety of noble metals and their alloys.

The bath consists of DMAB as a reducing agent, a suitable gold source and a stabilizer in a highly alkaline media consisting of KOH. The mechanism of electroless gold plating involves the reduction of the Au(I) to the elemental form accompanied by the simultaneous oxidation of DMAB. Electroless gold plating baths currently suffer from spontaneous bath decomposition or low plating rates under certain conditions of temperature and bath component concentrations. Industrial settings require that undesirable bath conditions be monitored and subsequently regulated to obtain maximum bath efficiency. Because the concentrations of bath components change continuously throughout the plating process, the implementation of a rapid, cost-effective means of online monitoring employing portable equipment is highly desirable.

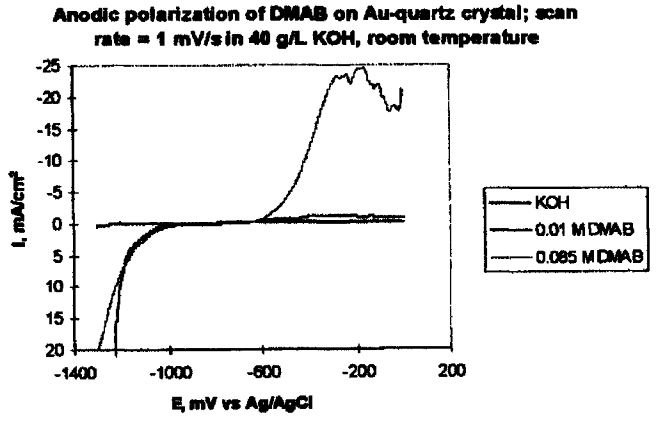

An adequate knowledge of the mechanism of DMAB oxidation and Au(I) reduction at the metal surface is integral to development of the on-line sensing system. We have investigated this mechanism using a variety of electrochemical techniques. This paper describes the development of on-line process monitors for the measurement of chemical compositions in the electroless gold plating bath using DMAB as reducing agent.

### Flow-injection chemiluminescence determination of captan in organized media using β-cyclodextrins

*J. V. García Mateo, M. C. Cheregi[1] and J. Martínez Calatayud[2], Colegio Universitario C.E.U., 'San Pablo', 46113 Moncada, Valencia, Spain; [1]University of Bucarest, Faculty of Chemistry, Panduri 9092 Bucharest, Romania; [2]Universidad de Valencia, Dpto. Quimica Analítica, 46100 Valencia, Spain*

β-Cyclodextrines (CDs) are very well-known cyclic oligosaccharides capable to form, with a wide variety of organic components, inclusion complexes which have numerous applications in pharmaceutical, food and environmental analysis. The inclusion of analyte molecules by CDs can offer interesting advantages. The structural conformation on the CD protects the fluorescing singlet state of analytes from external quenchers. As a consequence of inclusion complex formation, the rotation of the guest molecule is hindered and the relaxation of the solvent molecules is considerably decreased. Both of these effect can result in a decrease in the vibrational deactivation. This altered microenvironment can provide favourable conditions for enhanced quantum efficiencies and hence the intensities of luminescence.

The determination of captan was carried out using a FIA-manifold in which the pesticide dissolved in a β-cyclodextrine solution was injected into a carried stream of β-cyclodextrines organized medium to avoid the precipitation of the pesticide. Carrier then was merged with the oxidant and determined by direct chemiluminescence using a luminometer as detector. Five oxidizing systems; $MnO_4^-$, Ce(IV), $Fe(CN)_6^{3-}$, $H_2O_2$ and $H_2O_2 + OCl^-$ were tested for chemiluminescence. System $H_2O_2$ + $OCl^-$ was selected. The proposed FIA-manifold was applied to the determination of captan in commercial formulations and other environmental samples.

### Flow injection system with chemiluminometric detection for enzymatic determination of ascorbic acid

*Andrei F. Dănet, Mihaela Badea[1] and Hassan Y. Aboul-Enein[2], University of Bucharest, Faculty of Chemistry, PO Box 164, Bucharest 15, Romania; [1]Chemical Researches Institute, Department of Biotechnology, Splaiul Independentei 202, PO Box 67, Bucharest 15, Romania; [2]Bioanalytical and Drug Laboratory, Biological & Medical Research Department (MBC-03), King Faisal Specialist Hospital & Research Center, PO Box 3354, Riyadh, Saudi Arabia*

A simple, rapid and selective method for determination of ascorbic acid was developed by combining a FIA-system with a chemiluminometric detector and a reactor with L-ascorbate oxidase (Sigma) immobilized on controlled pore glass. We also used a FIA-system with dissolved enzyme. In the last case, as ascorbate oxidase we used a crude extract of cucumber (*Cucumis sativus*) made in phosphate buffer with a pH of 7.1, which contains 0.05% $NaN_3$.

We found that some reducing agents (e.g. ascorbic acid and mercaptoacetic acid) give chemiluminescence with luminol in the presence of hexacyanoferrate (III) in an alkaline solution. For enzymatic determination of ascorbic acid we used this new type of chemiluminescent reaction.

The reactor with ascorbate oxidase was used for selective decomposition of the ascorbic acid. The signal registered after the passage of the sample through the enzymatic reactor was compared with the one obtained for the sample that did not flow through the reactor. The difference between the two signals was dependent on the ascorbic acid concentration. The residual signal obtained after the decomposition of the ascorbic acid is given by the interference of foreign substances.

Several parameters that influenced the performance of the system were studied and discussed. The optimum parameters were: total flow rate 3 ml/min, length of the dispersion coil 40 cm, $pH_{sample}$ 5.5, luminol concentration 2 mM in sodium carbonate 0.1 M, ferricyanide concentration 20 mM. Different configurations of FIA-system were investigated in this paper.

The calibration graph was linear in the range 10–800 μM ascorbic acid. We applied this method for the determination of ascorbic in fruit juices.

### On-line LC-GC coupling—a new method for the determination of alkylphenols in environmental samples

*Hartmut Prast, Eva Goergens, Hans-Werner Dürrbeck, Klaus Günther and Friedhelm Rogies[1], Institut für angewandte Physikalische Chemie, Leo Brandt Straße, 52428 Jülich, Germany; [1]Gerstel GmbH, Aktienstraße 232–234, 45473 Mülheim an der Ruhr, Germany*

This paper describes an online LC-GC coupling system that allows fractions from an LC eluant stream to be transferred to a standard GC system, A large volume sampler equipped with a flow-cell takes a fraction of the eluant and introduces it into a PTV using the solvent venting/stop-flow technique. Sample volumes between 10 and 1000 µl can be injected.

It will be demonstrated that this system permits the determination of alkylphenolethoxylates (APEOs) and their degradation products, at ultra-trace levels in water, sludge and biological matrices.

Due to their surface active properties, alkylphenolethoxylates (e.g. nonylphenolethoxylates, APEOs) act as efficient surface cleaners, and represent an important class of non-ionic surfactants. They also have many industrial applications, e.g. flotation processes, paper production and in the production of pesticide formulations. In addition, APEOs have been extensively used in nearly all types of industrial and household detergents.

After introduction to the environment and subsequent treatment in sewage plants, the degradation products of APEO (e.g. alkylphenols, APs) are present in the aquatic environment.

The degradation products of APEOs are toxic, persistent, and due to a structural relationship to oestradiol-17β, oestrogenically active. In particular, the para-position of the phenolic OH-group and the branched alkyl chain are supposed to be decisive parameters for the pseudo-oestrogenic effect.

The effect is caused by the OH-group of the APs which is bonded to the hormone receptor replacing oestradiol-17β. Because not all AP-isomers show this pseudo-oestrogenic effect, the analytical separation and differentiation of these isomers is of extreme importance.

Problems related to the determination of alkylphenols in environmental samples are by-product and matrix interferences, low analyte concentration and losses during sample preparation. Neither LC nor GC analysis alone can solve these problems.

For the reasons listed above, a significant improvement in the determination of APs can be achieved through on-line coupling of these two different chromatographic techniques.

### On-line extraction of paclitaxel using fluorescence detection

*Mike Saleh, Mohamed Abdelnasser, Vincent Warren and Paul Kurtulik, Analytical Research and Development, Bristol-Myers Squibb, One Squibb Drive, New Brunswick, NJ 08903, USA*

Paclitaxel (BMS-181339-01) is a novel diterpene compound approved by the Food and Drug Administration (FDA) for the treatment of ovarian cancer. Paclitaxel is formulated as a 6 mg/ml solution in column-purified cremophor: ethanol (1:1 by volume) and marketed under the trade name TAXOL®. The presence of cremophor, a non-ionic polyoxyethylated castor oil in the formulation complicates the HPLC analysis. The major components of the column-purified cremophor interfere with some of the non-polar impurities/degradants of paclitaxel making it difficult to detect them using UV.

A gradient HPLC method has been developed to determine the impurity/degradant content of TAXOL® for injection concentrate prepared using semi-synthetic paclitaxel. The method incorporates UV detection for early-eluting impurities/degradants and fluorescence detection for late-eluting impurities/degradants, which could be obscured by co-eluting column-purified cremophor peaks using UV detection.

The method conditions as well as its applications are presented in this paper.

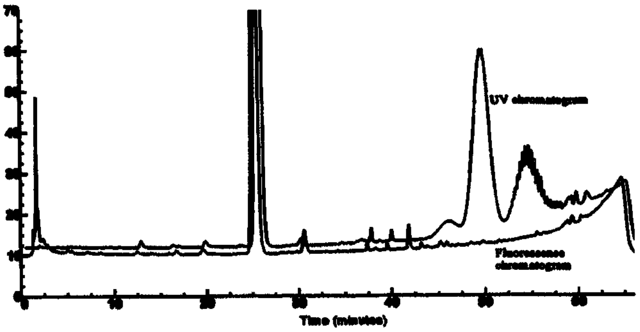

*Overlay of a TAXOL chromatogram from UV and fluorescence detectors.*

### Flow-injection methods for hydrochemical studies of marine environment

*Yury A. Zolotov, Lilia K. Shpigun and Patimat M. Kamilova, Institute of General & Inorganic Chemistry, Russian Academy of Sciences, Leninsky Prospect 31, Moscow 117907, Russia*

The hydrochemical study of the seawater column and pore water bottom sediments is a key component of marine environmental operation. In order to obtain reliable analytical data for chemical composition of these samples, a number of laboratory automation techniques have been proposed.

The aim of this paper is to demonstrate the applicability and advantages of flow injection analysis (FIA) with spectrophotometric and electrochemical detection to routine chemical determinations. The first demonstration will be in the area of inorganic trace element determinations based on FI spectrophotometric and electrochemical detection. Several types of FIA schemes will be presented for the purposes of dissolved orthophosphate, silicate, ammonium and sulphide ions in a wide concentration range, including a very low level (<1 µMol/l).

The second demonstration will be in the area of detecting trace levels of noble metal ions on chemically modified electrodes (CMES). In recent years, the use of CMES has begun to play an increasing role for FIA. However, the poor reproducibility of response over an extended period of time is a major uncontrolled variable which affects the performance of a carbon-paste CME. As one approach to alleviating this problem, we have prepared the macrocycle-modified silica matrixcomposite carbon electrodes. One such electrode with covalently immobilized dibenzo-16-tetrathiacrown-compound has been utilized as sensor in a FI scheme for anodic stripping voltammetric determining ultra-trace amounts of silver in seawater.

Finally, the shipboard applications of FIA for obtaining hydrochemical data (dissolved $O_2$, $CO_2$, $H_2S$, nutrients, etc.) during the cruises of the RN 'Academic Boris Petrov' will be presented in this paper. Current examples will be given where the new FI procedures used for investigation of the decomposition of organic matter in water mass and sediments in the Kara sea and Ob- and Yenesey estuaries as well as in the oxic-anoxic interface zone in the Black Sea.

### Total oxygen demand measurements for process control of COD in biosystem process samples

*Lynn W. Morlier, Monsanto Luling Plant, 12501 River Road, Luling, LA 70070, USA*

Total oxygen demand (TOD) measurements were evaluated for predictive correlation with the EPA-approved HACH method 8000 COD measurements, a 3-h test. Samples quantified were biosystem influent and effluent streams of carbon-, nitrogen- and phosphorus-containing compounds in varying amounts.

The TOD instrument, manufactured by IONICS, oxidizes the sample in a reaction chamber of platinum and high heat, and detects the oxygen consumed as the delta between the inlet and outlet oxygen stream. The method produces quantitative results in 4 min and was easy to calibrate and operate. Six months of data on the effluent sample streams and 2 months on the influent streams indicate that the correlation to COD is strong enough to use for process control purposes.

Besides the ability to obtain rapid results for control purposes, the method reduces hazardous waste associated with the disposal of reaction residue from the COD bichromate reduction.

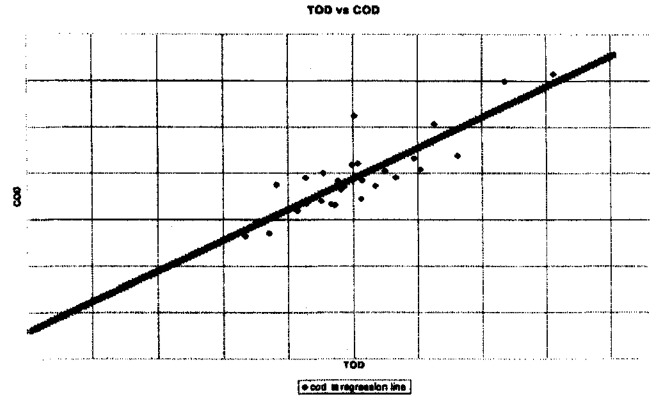

*Effluent data showing predicted COD values based on TOD measurements as represented by the regression line. Data points are the actual COD values obtained 3 h later.* $R^2 = 0.76$; *correlation sufficient for process control.*

### Determination of intact glucosinolates in Brassica vegetables by a rapid and reliable internal standard flow injection mass spectrometry method

*William C. K. Chiang and Rick E. A. Leitz, Rehnborg Center for Nutrition and Wellness, Nutrilite A Division of Amway, 19600 Sixth Street, Lakeview, CA 92567, USA*

Glucosinolates are important phytochemicals in Brassica vegetables. Their hydrolysis products, e.g. isothiocyanates, may play an important role in cancer prevention. Analytical methods for isothiocyanates are well established. However, current analytical methods for the parent glucosinolates tend to involve time-consuming sample preparation procedures and relatively lengthy analysis times. We report here a very rapid (<3 min), quantitative method for the analysis of glucosinolates in plant materials. This method utilizes an internal standard flow injection MS technique that overcomes the limitations of many of the older methods.

Because they are highly polar compounds, glucosinolates are readily separated by HPLC using ion pairing. However, the high concentration of ions in the mobile phase that is necessary to achieve good HPLC separation will interfere with subsequent on-line API-ESI MS analysis. On the other hand, conditions that are suitable for the identification of glucosinolates using API-ESI MS, do not provide adequate HPLC separation of multiple components. Our method utilizes API-ESI negative ion MS for the separation of the glucosinolates and the full scan and MS/MS capabilities of the mass spectrometer for glucosinolate identification.

Estimates of the glucosinolate content of vegetable samples are subject to inaccuracies during sample extraction either through physical losses or through hydrolysis via endogenous myrosinase enzymes. To avoid this limitation, our method employs the addition of an appropriate internal standard.

We have successfully applied the method to various Brassica vegetables including watercress, broccoli, nasturtium and peppercress for the quantitation of glucoraphanin, gluconasturtiin and glucotropaeolin.

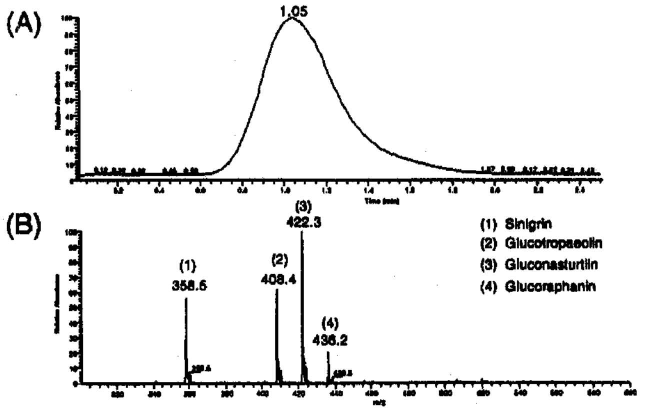

(A) *HPLC chromatogram of standard mixture.* (B) *MS spectrum of standard mixture.*

